# Sex Reversal in C57BL/6J XY Mice Caused by Increased Expression of Ovarian Genes and Insufficient Activation of the Testis Determining Pathway

**DOI:** 10.1371/journal.pgen.1002569

**Published:** 2012-04-05

**Authors:** Stephanie M. Correa, Linda L. Washburn, Ravi S. Kahlon, Michelle C. Musson, Gerrit J. Bouma, Eva M. Eicher, Kenneth H. Albrecht

**Affiliations:** 1 Department of Medicine, Biomedical Genetics, Boston University School of Medicine, Boston, Massachusetts, United States of America; 2 The Jackson Laboratory, Bar Harbor, Maine, United States of America; 3 Animal Reproduction and Biotechnology Laboratory, Department of Biomedical Sciences, Colorado State University, Fort Collins, Colorado, United States of America; Harvard Medical School, United States of America

## Abstract

Sex reversal can occur in XY humans with only a single functional *WT1* or *SF1* allele or a duplication of the chromosome region containing *WNT4*. In contrast, XY mice with only a single functional *Wt1*, *Sf1*, or *Wnt4* allele, or mice that over-express *Wnt4* from a transgene, reportedly are not sex-reversed. Because genetic background plays a critical role in testis differentiation, particularly in C57BL/6J (B6) mice, we tested the hypothesis that *Wt1*, *Sf1*, and *Wnt4* are dosage sensitive in B6 XY mice. We found that reduced *Wt1* or *Sf1* dosage in B6 XY^B6^ mice impaired testis differentiation, but no ovarian tissue developed. If, however, a Y^AKR^ chromosome replaced the Y^B6^ chromosome, these otherwise genetically identical B6 XY mice developed ovarian tissue. In contrast, reduced *Wnt4* dosage increased the amount of testicular tissue present in *Sf1*+/− B6 XY^AKR^, *Wt1*+/− B6 XY^AKR^, B6 XY^POS^, and B6 XY^AKR^ fetuses. We propose that *Wt1^B6^* and *Sf1^B6^* are hypomorphic alleles of testis-determining pathway genes and that *Wnt4^B6^* is a hypermorphic allele of an ovary-determining pathway gene. The latter hypothesis is supported by the finding that expression of *Wnt4* and four other genes in the ovary-determining pathway are elevated in normal B6 XX E12.5 ovaries. We propose that B6 mice are sensitive to XY sex reversal, at least in part, because they carry *Wt1^B6^* and/or *Sf1^B6^* alleles that compromise testis differentiation and a *Wnt4^B6^* allele that promotes ovary differentiation and thereby antagonizes testis differentiation. Addition of a “weak” *Sry* allele, such as the one on the Y^POS^ chromosome, to the sensitized B6 background results in inappropriate development of ovarian tissue. We conclude that *Wt1*, *Sf1*, and *Wnt4* are dosage-sensitive in mice, this dosage-sensitivity is genetic background-dependant, and the mouse strains described here are good models for the investigation of human dosage-sensitive XY sex reversal.

## Author Summary

It has been proposed that mice do not adequately model human disorders of sex development because testis determination is gene dosage-sensitive in humans, but initial studies suggested it is gene dosage-insensitive in mice. For example, XY humans with reduced functional *WT1* or *SF1* gene-dosage or increased *WNT4* gene-dosage can be sex reversed, whereas the equivalent XY mice were not sex-reversed on the genetic backgrounds previously reported. However, because testis determination in C57BL/6J mice is very sensitive to disruption, we tested the hypothesis that these genes are dosage-sensitive in C57BL/6J XY mice. We found that C57BL/6J-Y^AKR^ mice with reduced *Wt1* or *Sf1* gene-dosage were sex-reversed, whereas decreased *Wnt4* gene-dosage partially rescued testis development in four genetic systems where C57BL/6J XY mice develop ovarian tissue. Our results demonstrate that *Wt1*, *Sf1*, and *Wnt4* are dosage-sensitive in mice, that genetic background affects this sensitivity (as we suspect is true in humans), and that the mouse models described here are good models for human dosage-sensitive sex reversal. The results from these and other experiments lead to the hypothesis that the *Wt1* and/or *Sf1* alleles found in C57BL/6J mice are relatively “weak” testis-determining genes and their *Wnt4* allele is a hyperactive ovary-determining gene.

## Introduction

Testis differentiation in humans is gene dosage-sensitive. For example, heterozygosity for *WT1* or *SF1* (*NR5A1*) mutations and duplications containing *WNT4* has been associated with XY sex reversal (SR) [1]–[10]. Testis differentiation in humans also appears to be genetic background-sensitive because fathers who are heterozygous for SF1 mutations or hemizygous for SRY mutations can pass these mutations to their heterozygous/hemizygous XY daughters [4], [11], [12]. In contrast, initial studies in mice suggested testis differentiation is dosage-insensitive because heterozygosity for *Wt1* or *Sf1* null alleles or transgenic *Wnt4* over-expression did not cause XY SR [13]–[17]. This apparent human/mouse species difference led to the hypothesis that some genetic events leading to testis development differed between these species [18]. However, given the evidence that in mice genetic background plays a critical role in testis differentiation [19], [20], we examined the possibility that *Wt1*, *Sf1* and/or *Wnt4* would be haploinsufficient for normal testis determination on specific genetic backgrounds.

Work by our group and others has shown that testis determination in C57BL/6J (B6) inbred mice is particularly sensitive to genetic perturbation and a number of inherited gonadal sex reversal conditions have been identified in which B6 XY mice develop ovarian tissue [19]–[23]. The founding member of this group, B6-Y^POS^ sex reversal, occurs when the Y Chromosome (Chr) from *Mus domesticus poschiavinus* (Y^POS^) is transferred to the B6 genetic background, replacing the *Mus musculus*-derived Y^B6^ Chr [19]. B6 XY^POS^ fetal gonads develop as either ovotestes (gonads that contain both ovarian and testicular tissue) or ovaries, but not normal testes. The Y^POS^ Chr is not inherently defective, however, because it is testis-determining when transferred to most other inbred strain backgrounds, including DBA/2J (D2), BALB/cBy, C3H/HeJ and 129, or when present in F1 hybrids, such as in (D2×B6)F1 mice [24]–[27]. Furthermore, not all *M. domesticus*-derived Y Chrs (i.e., *Sry* alleles) behave like Y^POS^ (*Sry^POS^*) when transferred to B6. For example, testis differentiation in B6 XY mice containing the BUB/BnJ Y Chr appears to be normal whereas testis differentiation in B6 XY mice containing the AKR/J Y Chr is transiently delayed [28]. B6 XY^AKR^ mice do not develop permanent ovaries or ovotestes like B6 XY^POS^ mice. However, if B6 XY^AKR^ mice are heterozygous for either the *T^Orl^* or *T^hp^* deletion on Chr 17 they develop bilateral ovaries. In contrast, B6 XY^B6^ or B6 XY^BUB^ mice heterozygous for the *T^Orl^* deletion develop testes. Thus, the B6-Y^AKR^ consomic strain is more sensitive to XY sex reversal than the B6 strain. We previously showed that the transient delay in testicular cord differentiation is caused by the presence of particular SRY protein isoforms found in *M. domesticus* mice and that the reduced expression of some of these variants, such as the *Sry^POS^* allele, causes permanent sex reversal [29].

Further investigations revealed that B6-Y^POS^ sex reversal occurs because the *Sry* testis-determining gene present on the Y^POS^ Chr does not properly induce testis development if specific interacting genes, designated *tda* (testis determining autosomal) genes, are homozygous for B6 alleles. Two *tda* loci that differ between the B6 and D2 genomes and play a role in B6-Y^POS^ sex reversal were mapped to Chrs 2 (*tda2*) and 4 (*tda1*) [24]. Intriguingly, the *Wt1* (Wilms' tumor 1) and *Sf1* (steroidogenic factor 1, officially *Nr5a1*; nuclear receptor subfamily 5, group A, member 1, also known as *Ad4BP*, adrenal 4-binding protein) map to the *tda2* chromosomal region and *Wnt4* (wingless-related MMTV integration site 4) to the *tda1* region. Thus, we investigated *Wt1* and *Sf1* as candidates for *tda2*, and *Wnt4* as a candidate for *tda1*.

The *Wt1* and *Sf1* genes share a number of characteristics in addition to those noted above, some of which result from the fact that *Wt1* is a direct activator of *Sf1* expression [30] including: 1) both encode zinc finger transcription factors [31]–[33]; 2) both are expressed in XX and XY mouse urogenital ridges from about embryonic day (E) 9.5 until E12.5, when expression becomes sexually dimorphic and cell-type restricted; and 3) the gonad progenitor (i.e., genital ridge) is initially present in fetal mice that are homozygous for a null allele of either gene, but differentiation of the genital ridge does not progress and it regresses via apoptosis [14]–[17]. The *Wnt4* gene encodes a member of the WNT family of secreted signaling molecules that regulate cell-cell interactions during development. *Wnt4* is initially expressed in XX and XY genital ridges; thereafter expression is down-regulated in XY gonads but maintained in XX gonads [34]. Homozygosity for a *Wnt4* null mutation causes partial sex reversal of XX gonads resulting in the development of a testis-like coelomic vessel and the presence of cells expressing steroidogenic enzymes [34], [35]. In contrast, the gonadal phenotype in homozygous *Wnt4* null XY mice is relatively less severe; the gonads develop as testes, but the cords are initially fewer in number and disorganized [36]. To our knowledge, *Wnt4* heterozygotes have no reported gonad differentiation defects. In XY mice, transgenic *Wnt4* over-expression also causes testis differentiation defects but not sex reversal [13], [35].

To examine the possibility that the *Wt1*, *Sf1*, and *Wnt4* genes would be haploinsufficient on the B6 genetic background, we transferred null alleles to the B6 and D2 backgrounds and determined if reduced dosage influenced gonadal development in XY^B6^ and XY^AKR^ mice. Gonad morphology and marker gene expression in mutant versus normal fetal mice was analyzed using immunohistochemistry and confocal microscopy, RNA *in situ* hybridization, and quantitative RT-PCR. The results of these experiments indicated that testis differentiation was compromised at a very early stage and that *Sry* expression was significantly reduced in *Wt1*+/− or *Sf1+/−* B6 XY^AKR^ but not in *Wt1*+/− or *Sf1+/−* (D2×B6)F1 XY^AKR^ fetal gonads. We also found that testis development was mostly rescued in *Wt1*+/− and *Sf1+/−* B6 XY^AKR^ fetuses that contained two *Sry* alleles. These results are consistent with the hypothesis that the *Wt1^B6^* and *Sf1^B6^* alleles behave as hypomorphic alleles of testis-determining pathway genes in B6 XY mice if other genes in the genome are homozygous for the B6 allele. In contrast, reduced *Wnt4* dosage did not exacerbate the transient delay in testis differentiation in B6 XY^AKR^ gonads but rather appeared to ameliorate the phenotype, which suggests that the B6-derived *Wnt4* allele (*Wnt4^B6^*) is a hypermorphic allele of a gene in the ovary determination pathway. We also found that B6 *Wt1*+/− XY^AKR^, B6 *Sf1*+/− XY^AKR^, and B6 XY^POS^ fetuses with reduced *Wnt4* dosage developed more testicular tissue than *Wnt4*+/+ fetuses. These data are consistent with the hypothesis that *Wnt4^B6^* is an early functioning allele of an ovary-determining gene that antagonizes testis differentiation in XY mice. Expression of *Wnt4* and four other ovary-biased or ovary-specific genes was found to be elevated in E12.5 B6 vs. D2 XX ovaries. Together, our results suggest that B6 mice are sensitive to XY sex reversal compared to D2 mice because genes in the ovary determination pathway function earlier in the B6 genome than they do in the D2 genome.

The results reported here show that the *Wt1*, *Sf1*, and *Wnt4* genes are dosage-sensitive during mouse primary sex determination, as they are in humans, and that this effect is modulated by genetic background in the mouse, as is probably the case in humans. We propose that the dosage-sensitive C57BL/6 XY mouse strains described here are powerful models for further investigation of human dosage-sensitive XY sex reversals. In addition, our results support the candidacy of *Wt1*, *Sf1*, and *Wnt4* as *tda* genes, and suggest the testable hypothesis that B6 XY^POS^ sex reversal is cause by the additive effects of: 1) hypomorphic *Wt1^B6^* and *Sf1^B6^* alleles, and reduced activation of the testis differentiation pathway, 2) a hypermorphic *Wnt4^B6^* allele and hyperactivation of the ovary differentiation pathway, and 3) reduced function and expression of the *Sry^POS^* allele.

## Results

### *Wt1*+/− and *Sf1*+/− B6 XY^AKR^ mice are sex-reversed

To examine the possibility that *Wt1* and/or *Sf1* would be haploinsufficient on the B6 genetic background and to investigate their candidacy as *tda* genes, we transferred null alleles to the B6 and D2 backgrounds and determined if reduced dosage influenced gonadal development in XY^B6^ and XY^AKR^ mice. We reasoned that if the B6 alleles of *Wt1* and/or *Sf1* function as hypomorphs in the testis determination pathway, then further reducing their dosage in B6 XY mice should cause sex reversal, whereas reducing their dosage in D2 XY mice should not. Sex reversal was not evident in *Wt1*+/− or *Sf1*+/− B6 or D2 XY weanling or adult mice, and the mice were fertile. To increase the sensitivity of the genetic background to sex reversal, we mated *Wt1* +/− and *Sf1*+/− B6 XX females to B6 XY^AKR^ males. Based on the external sexual phenotype of 39 *Wt1*+/− B6 XY^AKR^ mice examined, 20 presented as male, 11 as hermaphrodite, and 8 as female (Table 1). In contrast, of the 20 *Sf1*+/− B6 XY^AKR^ offspring, none presented as male, 4 as hermaphrodite, and 16 as female. We noted a deficiency in the expected number of B6 *Sf1*+/− animals recovered, regardless of chromosomal sex. To test if these XY sex reversals were dependent on genetic background like B6-Y^POS^ sex reversal, offspring were analyzed from mating *Wt1*+/− B6 XX females to AKR/J XY^AKR^ males and *Sf1*+/− D2 XX females to B6 XY^AKR^ males. (D2 *Wt1*+/− mice were not available when these analyses were performed but similar results were obtained in the analysis of gonad morphology in E14.5–16 fetuses when mating *Wt1*+/− D2 XX females to B6 XY^AKR^ mice, Table 2.) In F1 hybrids, the heterozygous XY offspring presented as normal males and the number of *Sf1*+/− (D2×B6)F1 offspring recovered was Mendelian. We conclude that *Wt1*+/− and *Sf1*+/− B6 XY^AKR^ mice are sex reversed, *Sf1*+/− B6 mice have reduced viability, and both sex reversal and reduced viability are sensitive to genetic background. These results also indicate that the sex reversal phenotype is stronger in *Sf1* mutants because more *Sf1*+/− than *Wt1*+/− B6 XY^AKR^ mice presented as females and hermaphrodites.

**Table 1:** External sexual phenotype of *Wt1+/−* and *Sf1+/−* weaning age mice.

		XY pups				
Maternalgenotype	Paternalgenotype	+/−Females	+/−Hermaphrodites	+/−Males	Total +/−XY pups	TotalXY pups
B6 *Wt1*+/−	B6 +/+ XY^AKR^	8 (21%)	11 (28%)	20 (51%)	39	81
B6 *Wt1*+/−	AKR +/+ XY^AKR^	0 (0%)	0 (0%)	20 (100%)	20	47
B6 *Sf1*+/−	B6 +/+ XY^AKR^	16 (80%)	4 (20%)	0 (0%)	20	95^a^
D2 *Sf1*+/−	B6 +/+ XY^AKR^	0 (0%)	0 (0%)	13 (100%)	13	18

a Approximately 30% of B6 *Sf1+/−* pups die prior to weaning.

**Table 2:** Gonad phenotype in E14.5–16 *Wt1+/−* and *Sf1+/−* XY fetuses.

		E14.5–16 XY gonads				
Maternalgenotype	Paternalgenotype	+/−Ovaries	+/− Ovotestes	+/−Testes	+/− Abnormaltestes^a^	Total +/− XY gonads
B6 +/+	B6 *Wt1*+/− XY^B6^	0 (0%)	0 (0%)	28 (100%)	0 (0%)	28
B6 *Wt1*+/−	B6 +/+ XY^AKR^	5^b^ (10%)	23 (48%)	8 (17%)	12 (25%)	48
B6 *Wt1*+/−	D2 +/+ XY^AKR^	0 (0%)	0 (0%)	24 (100%)	0 (0%)	24
D2 *Wt1*+/−	B6 +/+ XY^AKR^	0 (0%)	0 (0%)	12 (100%)	0 (0%)	12
B6 +/+	B6 *Sf1*+/− XY^B6^	0 (0%)	0 (0%)	20 (100%)	0 (0%)	20
B6 *Sf1*+/−	B6 +/+ XY^AKR^	31 (91%)	3 (9%)	0 (0%)	0 (0%)	34
D2 *Sf1*+/−	B6 +/+ XY^AKR^	0 (0%)	0 (0%)	14 (100%)	0 (0%)	14

a Cord development is somewhat delayed at the anterior and/or posterior ends but the gonad is not an obvious ovotestis.

b Two were abnormally shaped but no testicular cords were evident.

Gonad morphology in E14.5–16 fetuses was examined because assessment of external sexual phenotype does not distinguish between primary and secondary sex reversal, and thus is only an estimate of the true sex reversal frequency. At this developmental stage small amounts of ovarian tissue are visually apparent in ovotestes and the initial delay in testicular cord development has resolved in B6 XY^AKR^ fetuses, thus preventing the misclassification of the gonad as an ovotestis. All *Wt1*+/− B6 XY^B6^, *Wt1*+/− (B6×D2)F1 XY^AKR^ and *Wt1*+/− (D2×B6)F1 XY^AKR^ gonads analyzed were testes (Figure S1 and Table 2). In contrast, approximately half of the 48 *Wt1*+/− B6 XY^AKR^ gonads analyzed were ovotestes (23) or ovaries (5), and half were normal (8) or abnormal testes (12). Similarly, all *Sf1*+/− B6 XY^B6^ and *Sf1*+/− (D2×B6)F1 XY^AKR^ gonads analyzed were testes, whereas the 33 *Sf1*+/− B6 XY^AKR^ gonads analyzed were ovotestes (3) or ovaries (31). These results are consistent with the weanling analysis and show that sex reversal is the result of primary (gonadal) sex reversal.

### Morphological and marker gene analysis of E13.5 *Wt1*+/− and *Sf1*+/− B6 XY^AKR^ gonads

**Figure 1:**
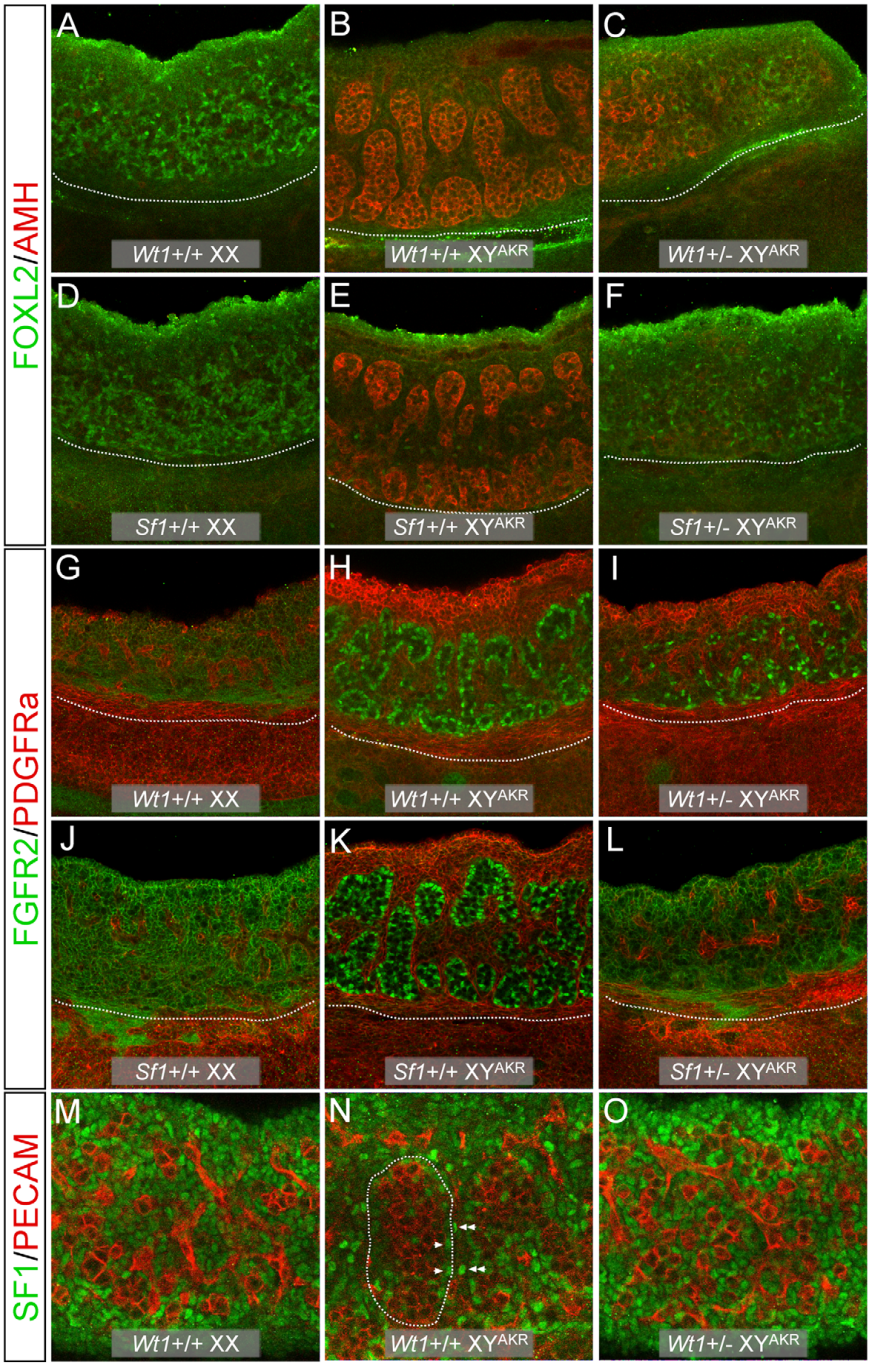
WIHC analysis of morphology and marker expression in *Wt1*+/− and *Sf1*+/− B6 XY^AKR^ E13.5 gonads. (A–L). Confocal images of gonad/mesonephros complexes with gonads shown above and mesonephroi below the white dotted line (20× magnification). Heterozygous XY gonads differentiated as ovotestes (C), ovaries (F, L, O), or gonads that were morphologically ovarian but expressed testicular markers (I). The *Wt1*+/− XY ovotestis in (C) expressed AMH, a Sertoli cell marker, in the central region containing testicular cords but not at poles that lacked cords. FOXL2, a granulosa cell marker, was expressed at the poles. FOXL2 also was expressed in isolated cells within regions containing testicular cords in B6 XY^AKR^ gonads (E). Heterozygous XY ovaries (F, L, O) expressed ovarian but not testicular markers. The *Wt1+/−* XY ovary in (F) expressed FOXL2 but not AMH throughout. The *Sf1*+/− XY ovary in (L) expressed FGFR2 on the surface of somatic cells (ovarian pattern) but did not have cells with nuclear localization of FGFR2 (testicular pattern, note localization in Sertoli cells (H, K)). The *Wt1* +/− XY gonad in (I) was ovarian morphologically but contained many cells with nuclear localization of FGFR2. These cells were not organized into testicular cords. PDGFRa was expressed at high levels in the coelomic epithelium of B6 XY^AKR^ gonads (H, K) and at lower levels in interstitial cells of B6 XX (G, J) and XY^AKR^ (H, K) gonads. Ovotestes, expressed PDGFRa at high levels in the coelomic epithelium of regions containing cords and at lower levels in regions without cords (data not shown). The heterozygous XY ovary in (L) expressed PDGFRa in a pattern similar to XX ovaries. The masculinized ovary in (I) expressed PDGFRa in a testis-like pattern. (M–O) Confocal images showing SF1 expression in gonads (40× magnification). At this stage, SF1 is expressed at fairly equal levels in all somatic cells in wild-type XX ovaries (M). In wild-type testes, expression is up-regulated in Sertoli cells (arrowheads) within testis cords (outlined with white dotted line) and in specific interstitial cells (double arrowheads), while it is down-regulated in most other interstitial cells (N). The expression of SF1 in *Wt1+/−* XY ovaries (O) resembled that of wild-type XX ovaries as expression was uniform in all somatic cells. Note that overall SF1 expression levels were similar in normal XX (M) vs. *Wt1*+/− XY (O) ovaries.

**Figure 2:**
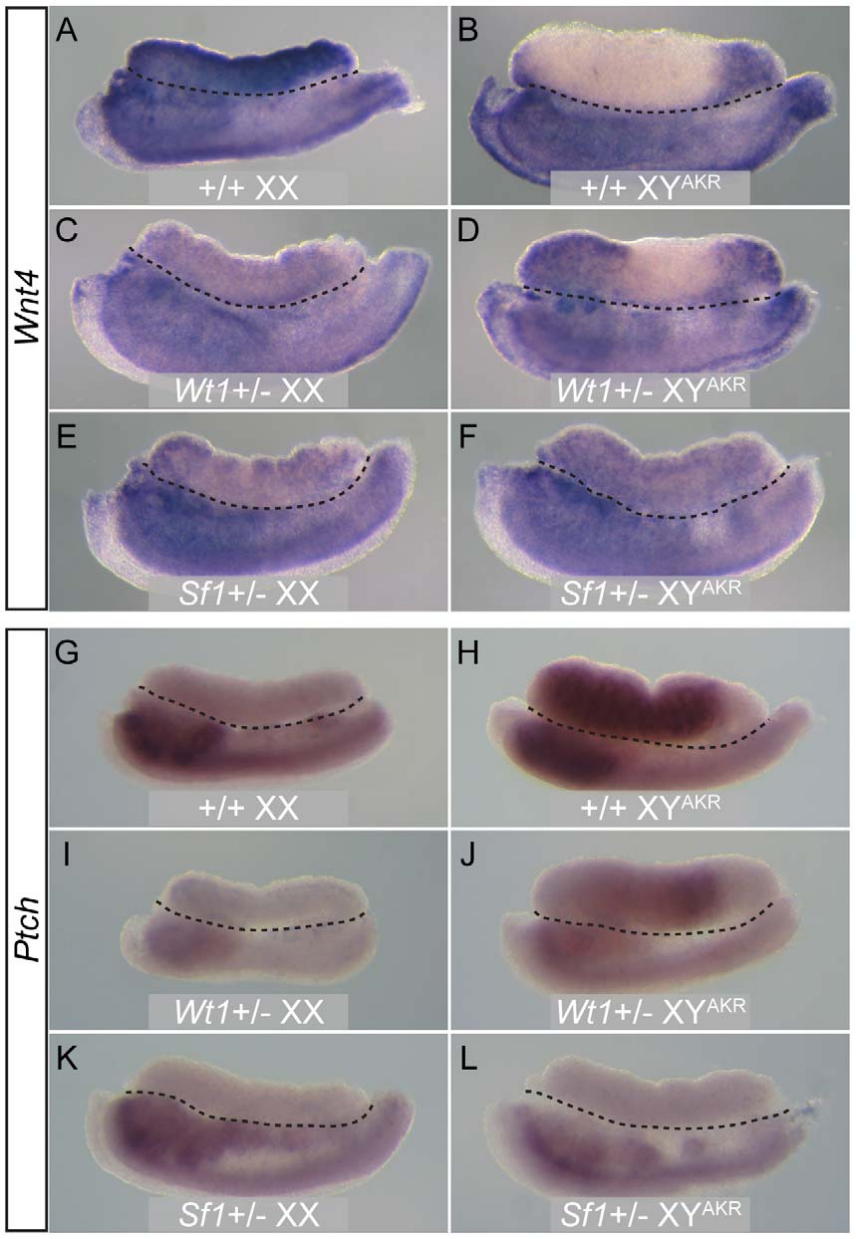
WISH analysis of the ovary somatic cell marker *Wnt4* and the testis Leydig cell marker *Ptch1* in E13.5 gonad/mesonephros complexes. *Wnt4* was expressed throughout +/+ XX ovaries, as expected (A), and at the anterior and/or posterior poles of +/+ B6 XY^AKR^ gonads in the regions without cords, but not in the central region containing cords (B). Expression was detected at the anterior and/or posterior poles of *Wt1*+/−(D) and *Sf1*+/− (data not shown) B6 XY^AKR^ ovotestes and throughout *Wt1*+/− (data not shown) and *Sf1*+/− B6 XY^AKR^ ovaries (F). *Wnt4* expression was reduced in B6 *Wt1*+/− XX (C) and *Sf1*+/− XX (E) ovaries compared to B6 *Wt1*+/+ XX (A) ovaries. Expression also was reduced in B6 *Wt1*+/− mesonephroi (C and D), but not in *Sf1*+/− mesonephroi (E and F). *Ptch1* expression was restricted to central regions containing testicular cords in +/+ B6 XY^AKR^ gonads (H) and was not detected in +/+ B6 XX ovaries (G), as expected. Expression was not detected in *Wt1*+/− and *Sf1*+/− B6 XX ovaries (I and K). *Ptch1* was expressed in the central regions of *Wt1*+/− (J) and *Sf1*+/− (data not shown) B6 XY^AKR^ ovotestes but at notably lower levels than in +/+ B6 XY^AKR^ gonads, and it was not expressed in *Wt1*+/− (data not shown) and *Sf1*+/− (L) XY^AKR^ ovaries. *Ptch1* expression was reduced in the anterior region of B6 *Wt1*+/− mesonephroi (I and J), but not in *Sf1*+/− mesonephroi (K and L). The gonad/mesonephros complex in each panel is oriented with the gonad above the mesonephros and separated by a dotted black line with the anterior (cranial) pole to the left. All images are at 10× magnification.

To gain further insight into the mechanism of sex reversal in *Wt1*+/− and *Sf1*+/− B6 XY^AKR^ gonads, whole-mount immunohistochemistry (WIHC, Figure 1) and whole-mount RNA *in situ* hybridization (WISH, Figure 2) were used to analyze morphology and marker gene expression in E13.5 gonads. Overall, the morphological analyses were consistent with the E14.5–16 analyses: *Wt1*+/− B6 XY^AKR^ gonads were classified as ovaries, ovotestes and testes and *Sf1*+/− B6 XY^AKR^ gonads were classified as ovaries and ovotestes. The *Wt1*+/− and *Sf1*+/− B6 XY^AKR^ gonads that developed as ovaries or ovotestes were phenotypically indistinguishable regardless of which gene was mutant. However, some *Wt1*+/− B6 XY^AKR^ gonads appeared to be very similar in morphology and marker gene expression pattern to those observed in control +/+ B6 XY^AKR^ gonads, whereas none of the *Sf1*+/− B6 XY^AKR^ gonads were phenotypically similar to +/+ B6 XY^AKR^ gonads.

Generally, *Wt1*+/− and *Sf1*+/− B6 XY^AKR^ gonads that developed as ovaries were morphologically indistinguishable from normal XX ovaries and strongly expressed ovary-specific markers, such as FOXL2 (Figure 1F), *Wnt4* (Figure 2F), *Fst* (data not shown) and *Bmp2* (data not shown) in a pattern similar to XX ovaries. Additionally, XY ovaries generally did not express testis-specific Sertoli cell markers, such as AMH (Figure 1F) or SOX9 (data not shown), or Leydig cell markers, such as *Ptch1* (Figure 2L), or markers such as GATA4, WT1 (data not shown) or FGFR2 (Figure 1L) in a testis-like pattern. However, about 20% of XY mutant gonads that had ovarian morphology contained cells that expressed Sertoli cell-specific markers, such as AMH or SOX9 (data not shown), or expressed markers, such as PDGFRa and FGFR2 in a testis-like pattern (Figure 1I). However, these cells were not organized in a pattern that resembled testicular cords. We conclude that Sertoli cell differentiation was initiated in some XY gonads that were morphologically ovarian, but was insufficient to initiate or sustain cord formation.

In *Wt1*+/− and *Sf1*+/− B6 XY^AKR^ ovotestes, testis markers generally were expressed in central regions containing cords whereas ovary markers were expressed at the anterior and posterior poles within regions lacking cords. For example, Figure 1C shows part of an ovotestis in which the central region contained cords and strongly expressed AMH, while the adjacent posterior region lacked cords and expressed FOXL2. Figure 2J shows an ovotestis that expressed *Ptch1* in the central region that contained testicular cords, but not in the ovarian regions at the anterior and posterior poles. [*Ptch1* expression in ovotestes was not as strong as that observed in B6 XY^AKR^ control gonads (compare Figure 2H and 2J)]. Conversely, Figure 2D shows an ovotestis expressing *Wnt4* in the anterior and posterior regions, but not in the central testicular region. Occasionally, testis- and ovary-specific markers were expressed in the same region (Figure 1E), where FOXL2 expression was detected in specific cells within a region containing well-formed testicular cords and strongly expressing the Sertoli cell marker AMH. We noted a few cells that appeared to express both AMH and FOXL2 (Figure S2A). However, because only whole-mount tissue was examined and because AMH is localized to the cell surface and FOXL2 to the nucleus, we were not able to exclude the possibility that the expression was in adjacent cells. Nevertheless, it is possible that some supporting cell progenitors can express both granulosa and Sertoli cell markers under certain conditions.

Because *Wt1* is functionally upstream of *Sf1* and is necessary for the initiation of *Sf1* expression [30], we examined SF1 expression in *Wt1*+/− B6 XY^AKR^ gonads using WIHC and found that expression levels appeared normal (Figure 1N–1O). Additionally, quantitative real-time RT-PCR (qRT-PCR) analysis did not detect a significant difference in *Sf1* expression in E12 gonads from *Wt1*+/− B6 XY^AKR^ vs. +/+ B6 XY^AKR^ fetuses (data not shown). On the other hand, *Wnt4* expression appeared to be reduced in *Wt1*+/− and *Sf1*+/− B6 XX ovaries compared to +/+ B6 XX ovaries, and *Ptch1* expression was strongly reduced in *Wt1*+/− B6 XX and B6 XY mesonephroi (Figure 2). Overall, the morphological and marker gene expression analyses showed that the testis determination pathway is aberrant at an early stage, that the ovarian pathway is activated in *Wt1*+/− and *Sf1*+/− B6 XY^AKR^ gonads, and that gene expression is affected in both XX and XY heterozygous gonad/mesonephros complexes.

A multigene qRT-PCR strategy was used to quantify gene expression changes in E12 +/− vs. +/+ B6 XY^AKR^ gonads, and E14 +/− ovaries vs. +/+ B6 XY^AKR^ testes (Table 3). These quantitative results were consistent with the morphological and marker gene analyses. For example, *Adamts19*, an ovary-specific gene, was significantly up-regulated in E14 *Wt1*+/− and *Sf1*+/− B6 XY^AKR^ ovaries vs. +/+ B6 XY^AKR^ control testes, and *Cyp11a1* and *Cyp17a1*, both Leydig cell specific genes, were expressed at significantly lower levels in *Wt1*+/− and *Sf1*+/− B6 XY^AKR^ ovaries.

**Table 3:** Quantitative RT–PCR analyses of E12 +/− versus +/+ B6 XY^AKR^ gonads and E14 +/− B6 XY^AKR^ ovaries (ovotestes excluded) versus +/+ B6 XY^AKR^ testes.

Stage	Gene	Fold change in *Wt1*+/−	Significant (p<0.05)
E12	*Bmp2*	2.4	T
	*Cbln4*	−5.4	G, T
	*Dhh*	−6.0	G, T
	*Mro*	−6.0	G, T
	*Ptgds*	−11.82	G, T
	*Defb19*	−3.4	T
E14	*Adamts19*	10.7	G, T
	*Bmp2*	10.7	G, T
	*Fgfr2*	2.4	G
	*Fst*	15.5	G, T
	*Sry*	9.6	G, T
	*Wnt4*	12.1	G, T
	*Aard*	−4.1	G, T
	*Amh*	−20.8	G, T
	*Cbln1*	−3.3	G, T
	*Cbln4*	−3.9	G, T
	*Col2a1*	−2.1	G
	*Col9a3*	−6.9	G, T
	*Cst9*	−5.1	G, T
	*Cyp11a1*	−4.7	G, T
	*Cyp17a1*	−8.2	G, T
	*Cyp26b1*	−4.0	G, T
	*Dhh*	−7.1	G, T
	*Etd*	−18.3	T
	*Hhip*	−4.1	G, T
	*Mro*	−2.1	G, T
	*Ptgds*	−8.0	G, T
	*Ren1*	−4.8	G, T
	*Sostdc1*	−4.7	G, T
	*Sox9*	−2.0	G, T
	*Defb19*	−8.6	G, T

Stage	Gene	Fold change in *Sf1*+/−	Significant (p<0.05)
E12	*Bmp2*	2.3	T
	*Sostdc1*	2.4	G, T
	*Aard*	−9.9	G, T
	*Amh*	−19.9	G, T
	*Cbln4*	−7.3	G
	*Cst9*	−22.6	G, T
	*Dax1*	−2	T
	*Dhh*	−13.1	G, T
	*Hsdb1*	−1.9	T
	*Ptgds*	−12.7	G, T
	*Sf1*	−2.1	G
	*StAR*	−1.9	T
	*Sox9*	−2.3	G
	*Defb19*	−8.7	T
E14	*Adamts19*	6.8	G, T
	*Bmp2*	7.2	G, T
	*Fgfr2*	2.9	G
	*Fst*	25.9	G, T
	*Sry*	56.9	G, T
	*Wnt4*	3.0	G
	*Aard*	−3.4	T
	*Amh*	−29.5	T
	*Cbln1*	−9.6	G, T
	*Cbln4*	−4.3	G, T
	*Col9a3*	−4.6	T
	*Cst9*	−7.3	T
	*Cyp11a1*	−66.9	T
	*Cyp17a1*	−89.9	T
	*Cyp26b1*	−8.3	T
	*Dhh*	−9.1	P = 0.06
	*Etd*	−22.3	G, T
	*Hhip*	−6.1	G, T
	*Hsdb1*	−14.7	G, T
	*Mro*	−2.4	G, T
	*Ptgds*	−15.4	G, T
	*Ren1*	−28.9	T
	*Scarb1*	−2.8	G, T
	*Sostdc1*	−8.2	G, T
	*Sox9*	−3.2	G, T
	*Defb19*	−18.5	G, T
	*Vnn1*	−2.6	G, T

G = GPR score equal or greater than 0.4.

T = Student's t-test.

### Ovary-specific markers are expressed at the anterior and posterior of B6 XY^AKR^ gonads

During analysis of marker gene expression in E13.5 *Wt1*+/− and *Sf1*+/− B6 XY^AKR^ gonads using WIHC and WISH we observed the expression of ovary-specific somatic cell markers in gonads from +/+ B6 XY^AKR^ fetuses. As expected, testis-specific markers, such as SOX9, AMH and *Ptch1*, were expressed in the central regions that contained testicular cords (Figure 1, Figure 2, and Figure S2). However, FOXL2, *Wnt4*, and *Fst* often were expressed in the anterior and posterior regions that lacked cords (Figure 1, Figure 2, Figure S2, and data not shown), indicating that the ovary differentiation pathway was activated in these regions. WISH analysis of the meiotic germ cell markers *Stra8* and *Rec8* [37] and the ovary-specific somatic cell marker *Irx3* [38] was used to determine if activation of the ovary differentiation pathway extended to the germ cells and to quantify the occurrence of ovary-specific marker gene expression (Figure S2B). We found that 11 of 13 B6 XY^AKR^ gonads clearly expressed either *Stra8* or *Rec8* at one or both poles and that 7 of 7 gonads expressed *Irx3* at both poles. In the 9 gonads where *Stra8* or *Rec8* were expressed at both poles, expression was higher at the anterior pole than the posterior pole, suggesting that the anterior-to-posterior wave of meiotic germ cell gene expression is maintained despite the presence of intervening testicular tissue [37]. It is important to note that testicular cords are present throughout B6 XY^AKR^ gonads after about E14.5. Therefore, these results are consistent with the idea that B6 XY^AKR^ gonads develop as transient ovotestes [39], [40].

### Testis and adrenal gland development is abnormal in B6 XY^B6^ fetuses that are double-heterozygous for *Wt1* and *Sf1*

**Figure 3:**
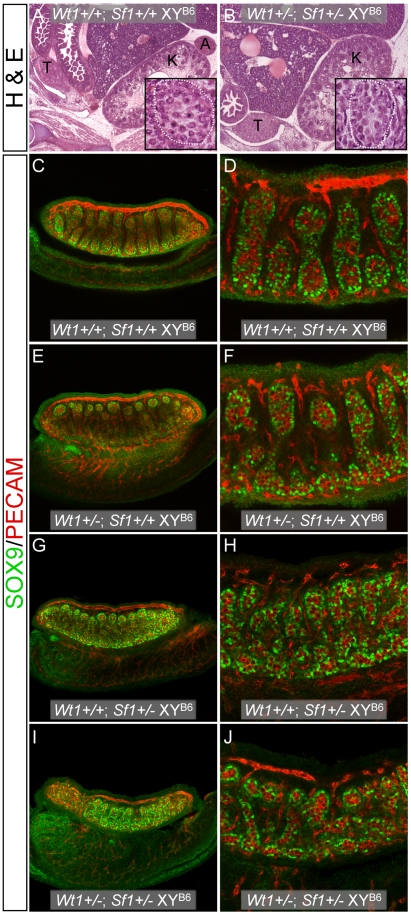
Histological and WIHC analysis of *Wt1*+/−; *Sf1*+/− B6 XY^B6^ fetuses. (A and B, 10× magnification) Hematoxylin and eosin staining of E14.5–15.5 fetal sagittal sections showed the absence of adrenal glands in *Wt1*+/−; *Sf1*+/− B6 XY^B6^ and XX (data not shown) fetuses. The inset panels in A and B are a magnified view showing that normal testicular cords were present in both *Wt1*+/+; *Sf1*+/+ and *Wt1*+/−; *Sf1*+/− B6 XY^B6^ fetuses (white dotted line outlines a section of a single cord). (C–J) WIHC analysis of E13.5 B6 XY^B6^ gonad/mesonephros complexes stained with SOX9 (Sertoli cells, green) and PECAM (germ cells and vascular endothelial cells, red). Panels C, E, G and I show 10× images taken with 0.7× zoom. Panels D, F, H, and J show 20× images. The gonads in all wild-type and single heterozygous E13.5 B6 XY^B6^ gonads were classified as testes, but testis cords in *Wt1*+/−; *Sf1*+/+ (E and F) and *Wt1*+/+; *Sf1*+/− (G and H), B6 XY^B6^ gonads were less developed and more disorganized than cords in *Wt1*+/+; *Sf1*+/+ (C and D) B6 XY^B6^ gonads. B6 *Wt1*+/−; *Sf1*+/+ gonads, however, were more similar to wild-type gonads than were *Wt1*+/+; *Sf1*+/− gonads. Testis differentiation was most affected in *Wt1*+/−; *Sf1*+/− B6 XY^B6^ gonads and these were classified as ovotestes: Sertoli cells in the anterior and posterior poles often expressed SOX9 but were not organized into cords (I and J).

Because the *Wt1*+/− and *Sf1*+/− B6 XY^AKR^ sex reversal phenotypes appear to be very similar and because *Wt1* is necessary for activation of *Sf1* expression in fetal gonads [30], we examined if B6 XY^B6^ mice were sex reversed when double heterozygous for *Wt1* and *Sf1* (*Wt1*+/−; *Sf1*+/−). We first attempted to ascertain the external sexual phenotype of the double heterozygotes at weaning. However, no *Wt1*+/−; *Sf1*+/− mice were recovered among the 84 progeny from B6 *Wt1*+/−×B6 *Sf1*+/− matings (Table 4). Because our previous results suggested that lethality could be sensitive to genetic background, progeny from B6 *Wt1*+/−×D2 *Sf1*+/− matings were analyzed. Among the 23 offspring were 4 *Wt1*+/−; *Sf1*+/− mice, indicating that lethality was rescued in (B6×D2)F1 hybrids. To determine at which developmental stage the lethality occurred, E14.5–15.5 fetuses from B6 *Wt1*+/−×B6 *Sf1*+/− matings were analyzed. Among the 12 offspring were 3 *Wt1*+/−; *Sf1*+/− mice (2 XY and 1 XX), indicating that lethality occurred between E15.5 and weaning. Histological examination of the three *Wt1*+/−; *Sf1*+/− fetuses showed that each had bilateral adrenal agenesis, which likely would cause neonatal lethality (Figure 3A and 3B). Both of the *Wt1*+/−; *Sf1*+/− XY fetuses had bilateral testes that appeared morphologically normal, but were smaller than those in wild-type XY littermates.

**Table 4:** Survival of *Wt1*+/−; *Sf1*+/− offspring.

			Offspring genotypes				
Stage	Maternalgenotype	PaternalGenotype	*Sf1*+/+;*Wt1+/+*	*Sf1*+/+;*Wt1+/−*	*Sf1*+/−;*Wt1+/+*	*Sf1*+/−;*Wt1+/−*	Total
Weaning	B6 *Wt1*+/−	B6 *Sf1*+/−	43	34	7	0	84
	B6 *Wt1*+/−	D2 *Sf1*+/−	3	4	2	3	12
E14.5–15.5	B6 *Wt1*+/−	B6 *Sf1*+/−	6	3	10	4	23

To extend these observations, gonad development was examined in E13.5 fetuses from B6 *Wt1*+/−×B6 *Sf1*+/− matings using WIHC (Figure 3C–3J). Of the 15 fetuses examined, 7 were *Wt1*+/−; *Sf1*+/−, which is consistent with the E14.5–15.5 survival data. Of the 9 XY^B6^ fetuses, 4 were *Wt1*+/−; *Sf1*+/−, 4 were *Wt1*+/+; *Sf1*+/−, and 1 was *Wt1*+/+; *Sf1*+/+. All 8 *Wt1*+/−; *Sf1*+/− XY gonads were classified as ovotestes, with large central testicular regions (Figure 3I and 3J). (Ovotestes in E13.5 double heterozygous fetuses appear to resolve into testes at later stages because the gonads in E14.5–15.5 double heterozygous fetuses do not contain ovarian tissue.) The 8 gonads from *Wt1*+/+; *Sf1*+/− XY fetuses were classified as testes but their cords appeared to be less well developed and more disorganized than +/+ B6 XY gonads (Figure 3). None of the XY offspring from these intercrosses were *Wt1*+/−; *Sf1*+/+, so we examined gonad development in E13.5 *Wt1*+/− B6 XY^B6^ fetuses generated from B6 *Wt1*+/−×B6 +/+ XY^B6^ crosses. All 6 gonads from *Wt1*+/− B6 XY^B6^ fetuses were classified as testes. However, their cords appeared to be less well-developed and more disorganized than normal B6 XY gonads: 4 displayed disorganized anterior and posterior poles and 2 had disorganized posterior poles (Figure 3E and 3F). In comparison, of the 6 gonads from +/+ B6 XY^B6^ fetuses, 2 had testicular cords throughout and 4 had disorganized posterior poles. In summary, the degree of testicular cord development in the E13.5 B6 XY^B6^ gonads could be ranked from most complete to least complete as: *Sf1*+/+; *Wt1*+/+>*Sf1*+/+; *Wt1*+/−>*Sf1*+/−; *Wt1*+/+>*Sf1*+/−; *Wt1*+/−. These results indicate that testis development is compromised in *Wt1*+/− and *Sf1*+/− B6 XY fetuses, even in the absence of the Y^AKR^ Chr, and that testis development is further compromised in mice that are double heterozygous for *Wt1* and *Sf1* null alleles.

### *Sry* expression is reduced in *Wt1*+/− and *Sf1*+/− B6 XY^AKR^ gonads and sex reversal is partially rescued by increased *Sry* dosage

**Figure 4:**
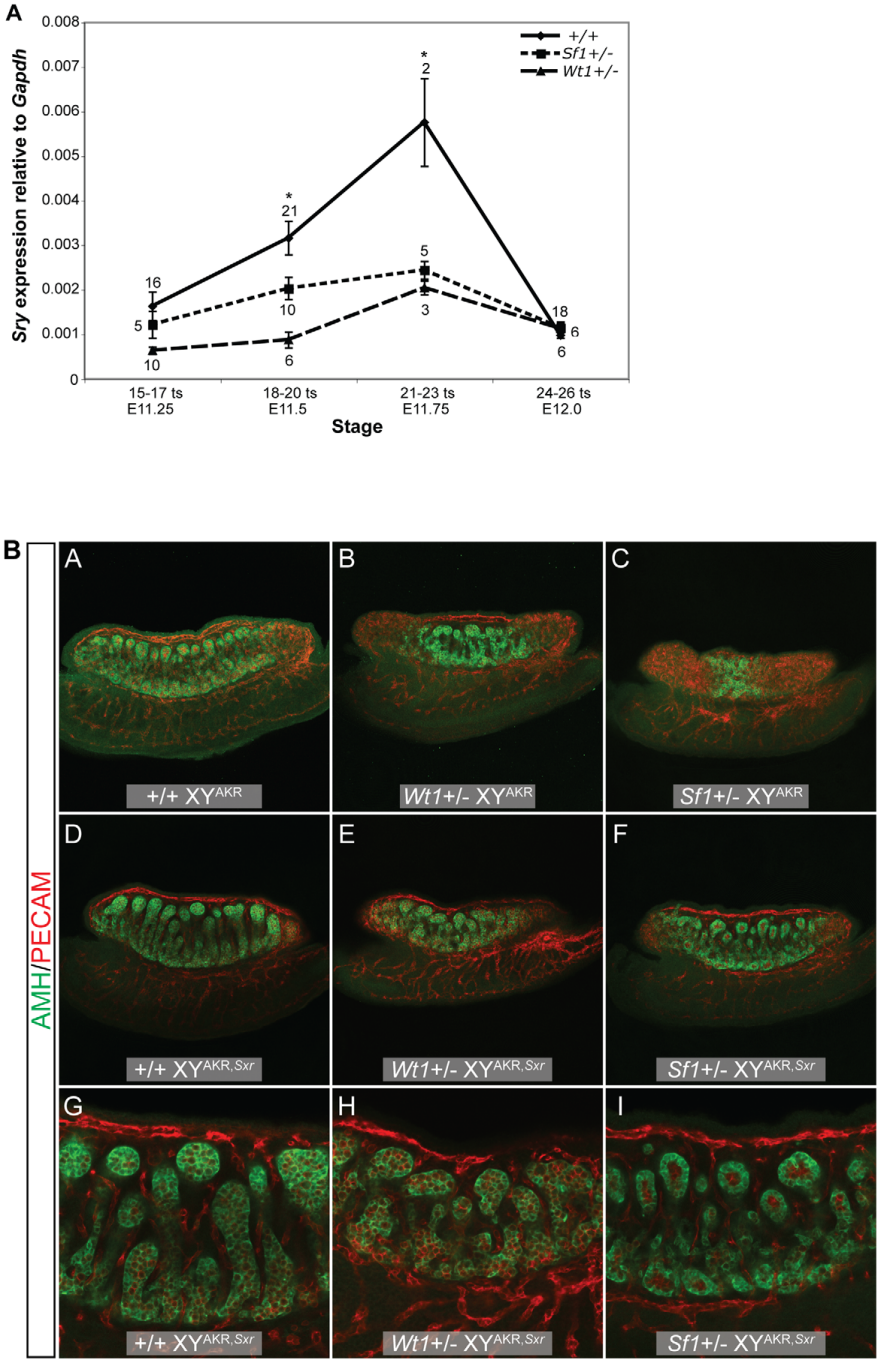
*Sry* expression is reduced in *Wt1*+/− and *Sf1*+/− B6 XY^AKR^ gonads, but increased *Sry* dosage did not fully restore normal cord differentiation. (A) Real-time qRT-PCR analysis of *Sry* expression relative to *Gapdh* (2^−ΔCt^) showing mean *Sry* transcript levels +/− standard error. The sample size for each group is shown adjacent to the corresponding data point. An asterisk denotes statistically significant effects of genotype (ANOVA, α = 0.05). There was a marginally significant effect of genotype at E11.25 (15–17 ts, ANOVA, F_2,28_ = 3.1670, p<0.0576) and a significant effect of genotype at E11.5 (18–20 ts, ANOVA, F_2,34_ = 6.999, p<0.0028) and E11.75 (21–23 ts, ANOVA, F_2,7_ = 15.41, p<0.0027). Pairwise comparisons revealed a significant reduction in *Sry* transcript levels in *Wt1+/−* vs. +/+ samples at E11.25 (15–17 ts, t_24_ = 2.224 , p<0.0179), E11.5 (18–20 ts, t_25_ = 3.653, p<0.0012), and in E11.75 (21–23 ts, t_3_ = 11.51, p<0.0014). Similarly, *Sry* expression was reduced in *Sf1+/−* vs. +/+ samples at E11.5 (18–20 ts, t_29_ = 2.125, p<0.0422) and E11.75 (21–23 ts, t_5_ = 6.142, p<0.0016). (B) The amount of tissue expressing the Sertoli cell marker AMH (green) was greater in *Wt1*+/− and *Sf1*+/− B6 XY^AKR,*Sxr*^ gonads (E and F), which have two copies of the *Sry* gene, compared to *Wt1*+/− and *Sf1*+/− B6 XY^AKR^ gonads (B and C), which have the normal single copy of the *Sry* gene (top two rows, 10× magnification with 0.7× zoom). However, while *Wt1*+/− and *Sf1*+/− B6 XY^AKR,*Sxr*^ gonads developed more testicular tissue than *Wt1*+/− and *Sf1*+/− B6 XY^AKR^ gonads, they consistently develop less testicular tissue than +/+ B6 XY^AKR,*Sxr*^ controls. Additionally, cords were less differentiated and less organized in heterozygous mutants when compared to wild-type B6 XY^AKR,*Sxr*^ gonads, even in the central regions of the gonads where cord differentiation was most complete (G–I, bottom row, 20× magnification). Germ and vascular endothelial cells are labeled by PECAM staining (red).

Because the morphological and marker gene expression analyses in E13.5 gonads suggested an early defect in the testis determination pathway, *Sry* expression in E11.5 *Wt1+/−* and *Sf1+/−* B6 XY^AKR^ vs. +/+ B6 XY^AKR^ gonad/mesonephros complexes was quantified. (*Sry* is not expressed in the mesonephros.) During peak *Sry* expression (E11.75), transcript levels in both mutants were less than 50% of the controls. We found that *Sry* transcript levels were reduced in *Wt1+/−* vs. +/+ samples at E11.25 (15–17 ts, t_24_ = 2.224 , p<0.0179), E11.5 (18–20 ts, t_25_ = 3.653, p<0.0012), and E11.75 (21–23 ts, t_3_ = 11.51, p<0.0014) but not significantly different at E12.0 (24–26 ts, Figure 4A). *Sry* transcript levels also were reduced in *Sf1+/−* vs. +/+ samples at E11.5 (18–20 ts, t_29_ = 2.125, p<0.0422) and E11.75 (21–23 ts, t_5_ = 6.142, p<0.0016), but were not significantly different at E11.25 (15–17 ts) or E12.0 (24–26 ts, Figure 4A). These results suggest that sex reversal in *Wt1+/−* and *Sf1+/−* B6 XY^AKR^ gonads is at least partially due to a failure to sufficiently up-regulate *Sry* expression and properly initiate the testis determination pathway. However, because pairwise comparisons between *Wt1+/−* vs. *Sf1+/−* samples did not reveal significant differences in *Sry* expression at any of the embryonic stages analyzed, differences in the level of *Sry* expression do not explain the differences in the severity of the *Wt1+/−* and *Sf1+/−* phenotypes.

To test the hypothesis that sex reversal in *Wt1+/−* and *Sf1+/−* B6 XY^AKR^ gonads is due, at least in part, to reduced *Sry* expression, gonad morphology was examined by WIHC in gonads from E13.5 fetuses that had increased *Sry* dosage due to the presence of the Y^AKR,*Sxr*^ Chr. [Classification was done independently by two investigators (SMC and KHA)]. This rearranged Y Chr carries two *Sry* alleles, one from the AKR/J inbred strain (*M. domesticus*) and one from the RIII inbred strain (*M. musculus*). Gonads in *Wt1+/−* and *Sf1+/−* B6 XY^AKR,*Sxr*^ fetuses consistently developed more testicular tissue than gonads in *Wt1+/−* and *Sf1+/−* B6 XY^AKR^ fetuses; however, it appeared that cord differentiation in the *Wt1+/−* and *Sf1+/−*B6 XY^AKR,*Sxr*^ gonads was not completely normal (Figure 4B, Table 5). Of the 8 *Wt1+/−* B6 XY^AKR,*Sxr*^ gonads analyzed, 7 were classified as abnormal testes because the testicular cords appeared to be more disorganized and less well developed than those in +/+ B6 XY^AKR,*Sxr*^ gonads. In fact, these cords appeared to be similar to the abnormal cords in *Wt1+/−* B6 XY^B6^ testes. One of the *Wt1+/−* B6 XY^AKR,*Sxr*^ gonads was classified as an ovotestis because it lacked cords in a small region at the posterior pole and had disorganized testicular cords in the remainder of the gonad. Of the 8 *Sf1+/−* B6 XY^AKR,Sxr^ gonads analyzed, 3 were classified as abnormal testes, with testicular cords throughout the gonad, and 5 were classified as ovotestes due to the absence of testicular cords at the posterior pole. The cords in these 8 gonads also were somewhat disorganized and less differentiated. In contrast, all 17 of the *+/+* B6 XY^AKR,*Sxr*^ gonads examined developed robust, well-organized testicular cords throughout the anterior, center and posterior regions. These data indicate that increasing *Sry* dosage in *Wt1+/−* and *Sf1+/−* B6 XY^AKR^ gonads by replacing the Y^AKR^ Chr with the Y^AKR,*Sxr*^ Chr dramatically increased the amount of testicular tissue that develops, but did not fully rescue cord differentiation.

**Figure 5:**
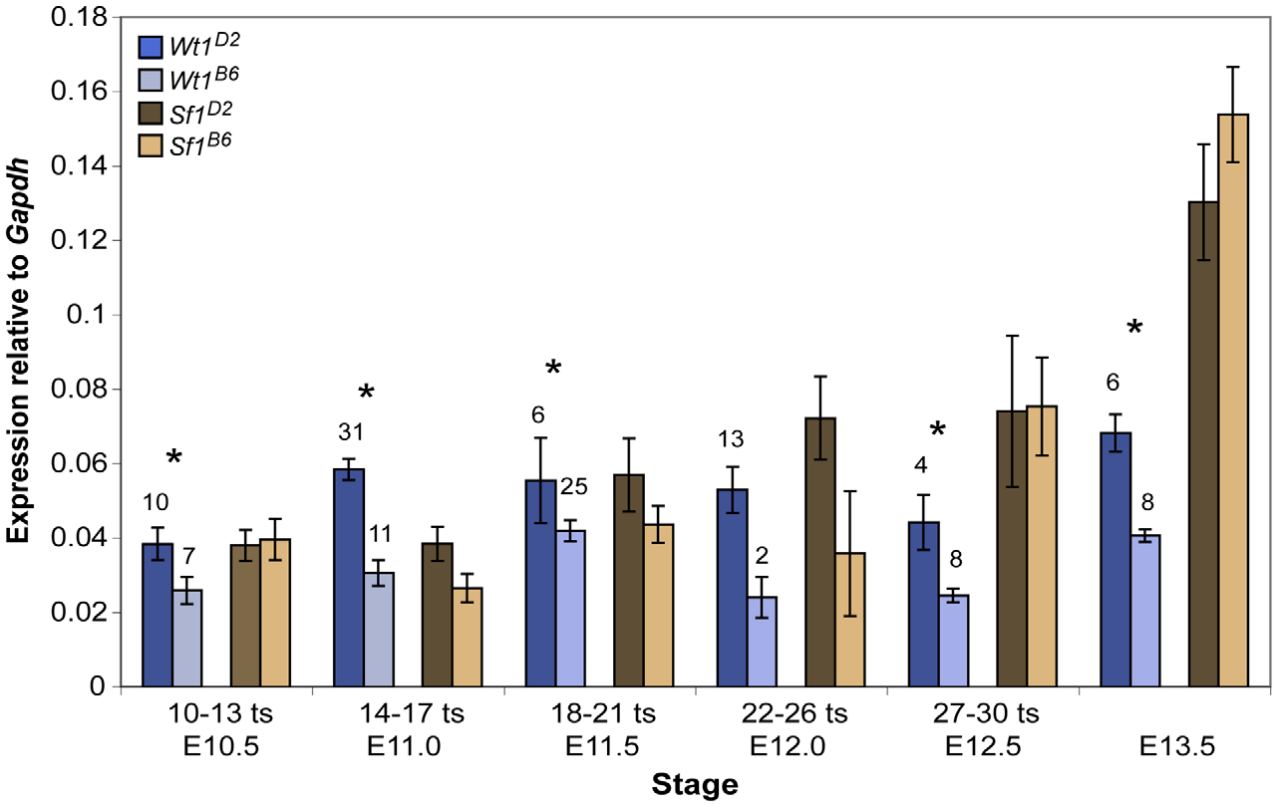
*Wt1* but not *Sf1* transcript levels were significantly lower in B6 versus D2 gonad/mesonephros complexes. Real-time qRT-PCR analysis of *Wt1* and *Sf1* expression normalized to *Gapdh* (2^−ΔCt^) showing mean transcript levels +/− standard error. *Wt1* and *Sf1* expression were determined in the same B6 and D2 samples, therefore the samples sizes are only shown above the corresponding error bars for the *Wt1^B6^* and *Wt1^D2^* comparisons. An asterisk denotes statistically significant differences in pairwise comparisons (α = 0.05). *Wt1* expression was significantly lower in B6 vs. D2 samples at E10.5 (10–13 ts, t_15_ = 2.063, p<0.0284), E11.0 (14–17 ts, t_40_ = 5.355, p<0.00002), E11.5 (18–21 ts, t_29_ = 1.699, p<0.0500), E12.5 (27–30 ts, t_10_ = 3.441, p<0.0032), and E13.5 (t_12_ = 5.985, p<0.0003). *Wt1* expression also was lower in B6 than in D2 samples at E12.0 (27–30 ts) but the difference did not reach statistical significance (t_13_ = 1.741, p<0.0526). *Sf1* expression was lower in B6 vs. D2 gonad/mesonephros complexes at E11.0 (t_40_ = 1.466, p<0.0753), E11.5 (t_29_ = 1.182, p<0.1235), and E12.0 (t_13_ = 1.208, p<0.1243), but these differences were not statistically significant.

**Table 5:** Increasing *Sry* dosage partially rescues the sex reversal phenotypes of E13.5 B6 XY^AKR^, *Wt1*+/− B6 XY^AKR^, and *Sf1*+/− B6XY^AKR^ gonads.

Genotype examined	Ovaries	Ovotestes	Abnormal testes	Testes	Total gonads
B6 *Wt1+/−* XY^AKR^	3 (25%)	7 (58%)	2 (17%)	0 (0%)	12
B6 *Wt1+/−* XY^AKR,*Sxr*^	0 (0%)	1 (13%)	7 (88%)	0 (0%)	8
B6 *Sf1+/−* XY^AKR^	21 (95%)	1 (5%)	0 (0%)	0 (0%)	22
B6 *Sf1+/−* XY^AKR,*Sxr*^	0 (0%)	5 (63%)	3 (38%)	0 (0%)	8
B6 +/+ XY^AKR,*Sxr*^	0 (0%)	0 (0%)	0 (0%)	17 (100%)	17

### The *Wt1* and *Sf1* B6 alleles require additional interacting genes to be homozygous for the B6 allele to function as hypomorphs

The results presented above suggest that the *Wt1^B6^* and *Sf1^B6^* alleles are hypomorphic alleles. One possibility is that the *Wt1^B6^* and *Sf1^B6^* alleles behave as intrinsic hypomorphs. An alternative possibility is that the *Wt1^B6^* and *Sf1^B6^* alleles function as hypomorphs only when one or more additional interacting gene(s) are homozygous for B6 alleles. To distinguish between these two alternatives, genetic crosses were designed that would generate hybrid (D2×B6)F1 XY^AKR^ mice that are heterozygous for the null (knockout) allele of *Wt1 or Sf1* and the *Wt1^B6^* or *Sf1^B6^* allele, respectively (e.g. *Wt1^KO^/Wt1^B6^* or *Sf1^KO^/Sf1^B6^* ) (Table 2). These F1 hybrid mice are genetically identical to *Wt1*+/− and *Sf1*+/− B6 XY^AKR^ mice at the *Wt1* and *Sf1* loci; however, the remainder of the genome is different because it is (D2×B6)F1 hybrid (except for the regions immediately around the *Sf1^KO^* and *Wt1^KO^* alleles that are derived from the 129 strain). Because *Wt1*+/− and *Sf1*+/− (D2×B6)F1 XY^AKR^ mice are not sex reversed, we conclude that the *Wt1^B6^* and *Sf1^B6^* alleles function as hypomorphs only when other genes (modifier genes) are homozygous for B6 alleles.

### Molecular analysis of the difference between the B6 and D2 alleles of the *Wt1* and *Sf1* genes

The B6 and D2 alleles of the *Wt1* and *Sf1* genes could encode different protein variants and/or have different expression patterns. A PCR primer-walking strategy using cDNA and genomic DNA as template was employed to determine the DNA sequence of the *Wt1* and *Sf1* genes present in the D2 genome and these sequences were compared to the mouse B6 reference sequence (data not shown). For *Wt1*, the ORF, 5′ and 3′ UTRs, and about 1 kb of the upstream flanking region were sequenced. One synonymous difference was identified in the ORF: a T (B6) vs. C (D2) substitution at the third position of codon 339, which was confirmed by its presence in dbSNP Build 128 (NCBI, rs27444886). There were no differences in the 5′ UTR and upstream flanking region. Four differences were identified in the 3′ UTR, two of which were confirmed by their presence in dbSNP (rs33129887 and rs13467742). Two single base pair differences in homopolymer length were not confirmed in dbSNP. The functional significance, if any, of the 3′ UTR polymorphisms is not readily apparent.

For *Sf1*, the ORF, 5′ and 3′ UTRs, and ∼1.5 kb of the upstream and 500 bp of the downstream flanking regions were sequenced. One synonymous and one non-synonymous difference were identified in the ORF. The synonymous polymorphism was a T (B6) vs. C (D2) substitution at the third position of the third codon. This polymorphism is represented in dbSNP (rs3142930) for certain inbred strains, but not for DBA/2J. The non-synonymous polymorphism was a G (B6) vs. T (D2) substitution in the first position of codon 172 resulting in an alanine (B6) vs. serine (D2) amino acid substitution and was confirmed by its presence in dbSNP (rs3142929). This polymorphism was independently discovered by Frigeri and colleagues who first identified it in ACTH resistant derivatives of the mouse Y1 adrenocortical tumor cell line [41]. There were no differences in the 5′ and 3′ UTRs. The upstream and downstream regions each had one difference, both in CA repeat units: The upstream CAAA repeat had 6 units in B6 vs. 5 units in D2, while the downstream CA repeat had 17 units in B6 vs. 22 units in D2. Neither polymorphism is present in dbSNP and their functional significance is not readily apparent.

To gain insight into whether the presence of the *Sf1^A172^* (B6) vs. *Sf1^S172^* (D2) allele was correlated with B6 XY sex reversal, and thus of potential functional significance, we took advantage of the fact that when females from different inbred strains are mated to B6 XY^POS^ hermaphrodites the F1 XY offspring are either sex reversed or normal, and thus the inbred strains can be classified as B6-like or D2-like, respectively (Table 6). Using this assay we identified two strains (C57BL/10J and NZB/BlNJ) that are B6-like and five strains (129S1/SvImJ, C3H/HeSnJ, SM/J C58/J and BALB/cBy) that are D2-like. A PCR-RFLP genotyping assay that was developed by Frigeri and colleagues [41] was used to determine the strain distribution pattern of the *Sf1^A172^* and *Sf1^S172^* alleles among these seven strains. As shown in Table 6, two strains were phenotypically and genotypically discordant. NZB/BlNJ was B6-like phenotypically but it has an *Sf1^S172^* allele, and BALB/cBy was D2-like phenotypically but it has an *Sf1^A172^* allele. We thus conclude that the presence of an *Sf1^A172^* vs. *Sf1^S172^* allele alone is not strictly correlated with B6 XY sex reversal.

**Table 6:** Strain distribution pattern of the *Sf1^A172^* versus *Sf1^S172^* alleles.

Inbred strain	F1 phenotype	*Nco*I genotype	rs3142929 confirmed
C57BL/6J	B6-like	+	+
C57BL/10J	B6-like	+	−*
NZB/BlNJ	B6-like	−	+
DBA/2J	D2-like	−	+
129S1/SvImJ	D2-like	−	+
C3H/HeSnJ	D2-like	−	not available
SM/J	D2-like	−	+
C58/J	D2-like	−	+*
BALB/cBy	D2-like	+	+

*: Indicates an imputed value.

The *Wt1* gene encodes multiple splice isoforms. Of particular interest here, alternative splicing of exon nine gives rise to two protein isoforms that differ by three amino acids and are referred to as the +KTS and −KTS isoforms. Both isoforms and their correct relative expression are necessary for normal gonad development [42]. For example, Frasier syndrome in humans is associated with XY sex reversal and caused by reduced expression of the *Wt1* +KTS isoform [43]. Thus, it was possible that B6 XY^AKR^ *Wt1*+/− sex reversal could be caused by altered +KTS/−KTS isoform ratios and qRT-PCR using primers that span the exon nine splice junction was employed to examine if the ratio of +KTS/−KTS splice isoforms is different in E10.5–14.5 B6 vs. D2 XX and XY gonad/mesonephros complexes. We found that the relative expression of the *Wt1* +KTS and −KTS isoforms did not differ between B6 and D2 gonad/mesonephros complexes (data not shown).

To test the hypothesis that the *Wt1^B6^* and *Sf1^B6^* alleles function as hypomorphs because they are expressed at lower levels or with different temporal profiles than the *Wt1^D2^* and *Sf1^D2^* alleles, respectively, transcript levels in B6 vs. D2 XY gonad/mesonephros complexes from E10.5–13.5 fetuses were quantified using qRT-PCR (Figure 5). *Wt1* expression was significantly lower in B6 vs. D2 samples at all stages. *Sf1* expression also was lower in B6 vs. D2 gonad/mesonephros complexes from E11.0–12.0 fetuses, but these differences were not statistically significant. These *Wt1* and *Sf1* expression results were corroborated in XX samples from similar stages, and the *Sf1* expression results were further confirmed by using an independent SYBR green qRT-PCR assay to analyze a subset of the samples (data not shown). In summary, these molecular analyses suggest that *Wt1^B6^* is a hypomorph due to reduced expression and that *Sf1^B6^* is a hypomorph possibly due to altered protein structure and/or the presence of other modifier B6 loci.

### Reducing *Wnt4* dosage increases the amount of testis tissue that develops in multiple cases of B6 XY sex reversal

**Figure 6:**
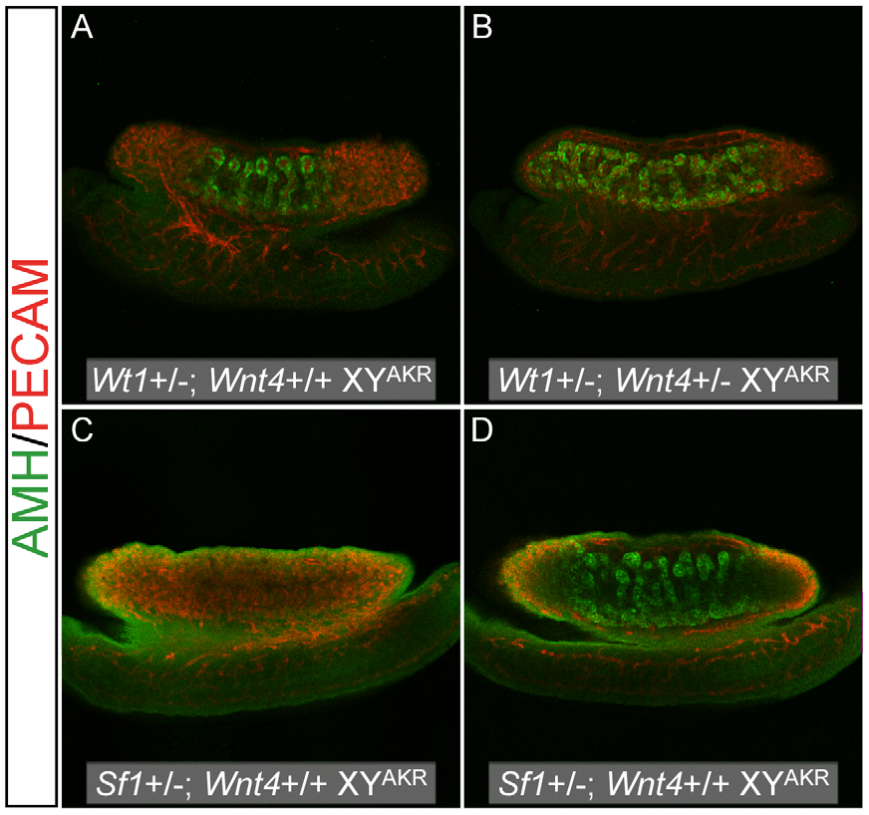
Reduced *Wnt4* dosage partially rescued cord differentiation in B6 XY genotypes that typically develop ovarian tissue. Representative images are shown for *Wt1*+/− (A and B) and *Sf1*+/− (C and D) B6 XY^AKR^ E13.5 gonads that were either *Wnt4*+/+ (A and C) or *Wnt4*+/− (B and D). The amount of tissue expressing the Sertoli cell marker AMH (green) was consistently greater in *Wnt4*+/− vs. *Wnt4*+/+ gonads for all four sex reversals examined. Germ and vascular endothelial cells are labeled by PECAM staining (red).

To examine the possibility that *Wnt4* would be haploinsufficient on the B6 genetic background and to investigate its candidacy as a *tda* gene, we transferred a *Wnt4* null allele onto the B6 genetic background and assessed if *Wnt4* heterozygosity exacerbated XY sex reversal in B6 XY^POS^, B6 XY^AKR^, *Sf1*+/− B6 XY^AKR^, and *Wt1*+/− B6 XY^AKR^ fetuses. WIHC was used to examine gonad morphology and marker gene expression at E13.5. Each gonad/mesonephros complex was assessed at dissection for the presence of cords and male-specific vasculature, and classified as an ovary, ovotestis, or testis (Table 7). Classification was done prior to ascertainment of the fetal genotype and was done by a single investigator (SMC) for consistency. A subset of complexes of each genotype was processed for WIHC and examined by two investigators (SMC and KHA) to confirm the initial classification (Figure 6). In all four experimental groups, reducing *Wnt4* dosage increased the area containing testicular cords and reduced the area without obvious structure in XY gonads. In B6 XY^POS^ fetuses, 5 of the 12 gonads analyzed from *Wnt4+/+* fetuses were classified as ovaries and none were classified as testes, whereas 7 of the 28 gonads analyzed from *Wnt4+/−* fetuses were classified as ovaries and 4 were classified as testes. These data indicate that reducing *Wnt4* dosage in B6 XY^POS^ fetuses decreased the percentage of ovaries from 42% to 25% and increased the percentage of testes from 0% to 14%. In B6 XY^AKR^ fetuses, 12 of the 16 gonads analyzed from *Wnt4*+/+ fetuses were classified as ovotestes and 4 were classified as testes, whereas 6 of the 18 gonads analyzed from *Wnt4*+/−fetuses were classified as ovotestes and 12 were classified as testes. Thus, reducing *Wnt4* dosage in B6 XY^AKR^ mice decreased the percentage of ovotestes from 75% to 33% and increased the percentage of testes from 25% to 67%. In B6 *Wt1*+/−XY^AKR^ fetuses, 3 of the 12 gonads analyzed from *Wnt4+/+* fetuses were classified as ovaries, 5 as ovotestes, and 4 as testes, whereas none of the 6 gonads analyzed in *Wnt4+/−* fetuses were classified as ovaries, 1 was an ovotestis and 5 were testes. Thus, reducing *Wnt4* dosage in *Wt1*+/− B6 XY^AKR^ mice decreased the percentage of ovaries from 25% to 0%, decreased the percentage of ovotestes from 42% to 17%, and increased the percentage of testes from 33% to 83%. In *Sf1*+/− B6 XY^AKR^ fetuses, all 8 gonads analyzed from *Wnt4+/+* fetuses were classified as ovaries, whereas 2 of the 12 gonads analyzed from *Wnt4+/−* fetuses were classified as ovaries, 3 as ovotestes, and 7 as testes. Thus reducing *Wnt4* dosage in B6 *Sf1*+/− XY^AKR^ mice decreased the percentage of ovaries from 100% to 17%, increased the percentage of ovotestes from 0% to 25%, and increased the percentage of testes from 0% to 58%. The WIHC analyses confirmed these results (Figure 6).

**Table 7:** Reducing the *Wnt4* dosage partially rescues the sex reversal phenotypes of E13.5 B6 XY^POS^, B6 XY^AKR^, B6 *Wt1*+/− XY^AKR^, and B6 *Sf1*+/− XY^AKR^ gonads.

			E13.5 XY gonad phenotype			
Maternalgenotype	Paternalgenotype	Fetal genotype	Ovaries	Ovotestes	Testes	Total gonads
B6 *Wnt4+/−*	B6 XY^POS^	B6 *Wnt4+/+* XY^POS^	5 (42%)	7 (58%)	0 (0%)	12
		B6 *Wnt4+/−* XY^POS^	7 (25%)	17 (61%)	4 (14%)	28
B6 *Wnt4+/−*	B6 XY^AKR^	B6 *Wnt4+/+* XY^AKR^	0 (0%)	12 (75%)	4 (25%)	16
		B6 *Wnt4+/−* XY^AKR^	0 (0%)	6 (33%)	12 (67%)	18
B6 *Wt1+/−*	B6 *Wnt4+/−* XY^AKR^	B6 *Wt1+/−; Wnt4+/+* XY^AKR^	3 (25%)	5 (42%)	4 (33%)	12
		B6 *Wt1+/−; Wnt4+/−* XY^AKR^	0 (0%)	1 (17%)	5 (83%)	6
B6 *Sf1+/−*	B6 *Wnt4+/−* XY^AKR^	B6 *Sf1+/−; Wnt4+/+* XY^AKR^	8 (100%)	0 (0%)	0 (0%)	8
		B6 *Sf1+/−; Wnt4+/−* XY^AKR^	2 (17%)	3 (25%)	7 (58%)	12

These data indicate that *Wnt4^B6^* acts like an anti-testis gene. Using qRT-PCR we found that *Sry* expression in *Wnt4*+/+ vs. *Wnt4*+/− B6 XY^AKR^ was not significantly different during peak *Sry* expression in E11.75 (21–23 ts) gonad/mesonephros complexes (data not shown), suggesting that *Wnt4^B6^* compromises the testis pathway downstream of *Sry* [36]. Together, these data suggest that *Wnt4^B6^* behaves as a hypermorphic allele of a gene in the ovary determination pathway, rather than a hypomorphic allele of a gene in the testis determination pathway.

### Elevated expression of ovary-biased genes in B6 versus D2 XX ovaries

**Figure 7:**
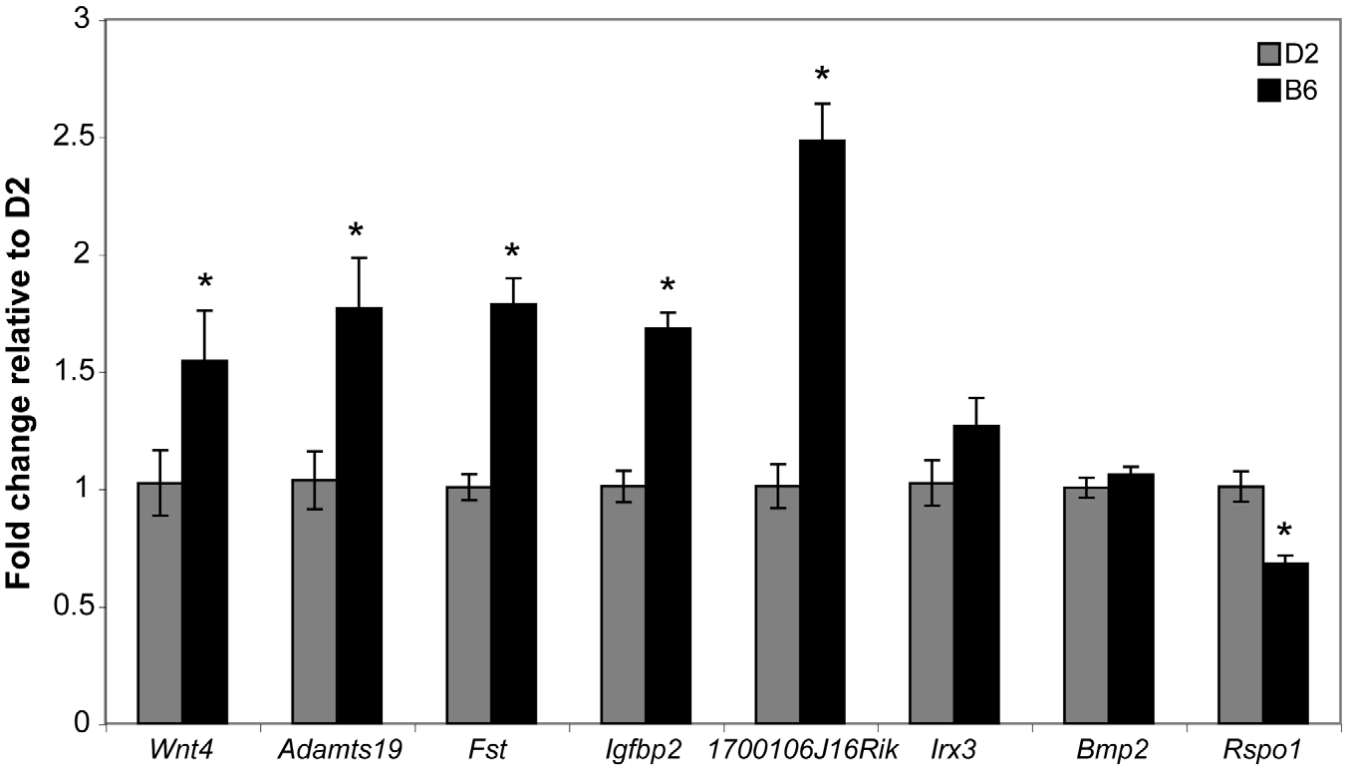
Real-time qRT–PCR analysis of ovarian marker gene expression in B6 versus D2 E12.5 XX gonads. Expression of ovary-specific genes *Wnt4*, *Adamts19*, *Fst*, *Ifgbp2*, *1700106J16Rik*, *Irx3*, *Bmp2*, and *Rspo1* were measured in the same samples and normalized to *Hprt* (D2, n = 6 gonad/mesonephros complexes; B6, n = 10 gonad/mesonephros complexes). Fold change relative to mean D2 values was determined using the 2^−ΔΔCt^ method and plotted as the average per sample group +/− standard error. An asterisk denotes statistically significant differences in pairwise comparisons (α = 0.05). The expression levels of *Wnt4* (t_14_ = 1.938, p<0.0365), *Adamts19* (t_14_ = 2.13, p<0.0257), *Fst* (t_14_ = 4.865, p<0.0001), *Ifgbp2* (t_14_ = 4.987, p<0.0001), and *1700106J16Rik* (t_14_ = 5.002, p<0.0001) were significantly higher in B6 vs. D2 XX ovaries. Expression of *Irx3* and *Bmp2* did not significantly differ between the two strains, and *Rspo1* expression was significantly lower in B6 vs. D2 XX ovaries (t_14_ = 4.612, p<0.0002).

The *Wnt4* results presented above suggested the possibility that the ovary differentiation pathway is prematurely- or hyper-activated in B6 gonads, thereby suppressing the testis determination pathway. As with *Wt1* and *Sf1*, the B6 and D2 *Wnt4* alleles could encode different protein variants or have different expression patterns. We determined the DNA sequence of the *Wnt4* ORF present in the D2 genome and found that it did not differ from that in B6, which was confirmed by data present in dbSNP Build 128 (data not shown). We used qRT-PCR to compare the expression of *Wnt4* and seven other genes in the ovary differentiation pathway in E12.5 B6 vs. D2 XX ovaries to test the hypothesis that *Wnt4^B6^* is an expression hypermorph and to investigate the possibility that the ovary differentiation pathway is prematurely- or hyper-activated in B6 gonads (Figure 7). The seven genes were chosen because their expression normally is ovary-biased at E12.5 (i.e., their expression is either ovary-specific or higher in ovaries vs. testes) [34], [38], [44]–[46]. We found that the expression of *Wnt4* (p<0.0004), *Adamts19* (p<0.026), *Fst* (p<0.0001), *Igfbp2* (p<0.0001), and *1700106J16Rik* (p<0.0001) was significantly higher in B6 vs. D2 ovaries; that expression of *Irx3* (p<0.177) and *Bmp2* (p<0.323) was not significantly different; and that expression of *Rspo1* (p<0.0002) was significantly lower. These results demonstrate that *Wnt4* and other genes known, or thought to be, in the ovary differentiation pathway are hyperactivated in E12.5 B6 vs. D2 XX gonads.

## Discussion

It has been suggested that some genetic events leading to testis determination differ between humans and mice because several genes that are haploinsufficient in humans appeared to be haplosufficient in mice [18]. However, based on our studies of B6-Y^POS^ sex reversal we hypothesized that genetic background would have a significant influence and that some of these genes would be haploinsufficient on the B6 genetic background. We chose to analyze the *Wt1*, *Sf1*, and *Wnt4* genes for two main reasons: 1) they are dosage-sensitive in humans but initial studies suggested they were dosage-insensitive in mice, and 2) they are good candidates for *tda* genes based on genetic location and because gonad differentiation is abnormal in homozygous knockout mice. Our analyses show that these three genes can be dosage-sensitive in mice, depending on the genetic background, and provide insights into the mechanisms of B6 XY sex reversal.

### The *Wt1* and *Sf1* genes are dosage-sensitive on the B6 genetic background

Our results show that the *Wt1* and *Sf1* B6 alleles are haploinsufficient for normal testis differentiation when present on the B6 genetic background. We found that *Wt1*+/− and *Sf1*+/−B6 XY^B6^ mice developed as males; however, testis differentiation was transiently delayed and was further compromised in double heterozygous *Wt1*+/−; *Sf1*+/− B6 XY^B6^ fetuses. These data are consistent with evidence that *Wt1* activates *Sf1* transcription [30]. When the genetic background was further sensitized by substituting the Y^B6^ Chr with the Y^AKR^ Chr, testis differentiation was more severely affected and *Wt1*+/− and *Sf1*+/− B6 XY^AKR^ mice developed ovotestes and ovaries. In contrast, all *Wt1*+/− and *Sf1*+/− (D2×B6)F1 XY^AKR^ mice developed testes. Taken together, these results show that the *Wt1* and *Sf1* B6 alleles are hypomorphic relative to the D2 alleles. Analysis of morphology and marker gene expression in E13.5 *Wt1*+/− and *Sf1*+/− B6 XY^AKR^ gonads showed that some developed as ovaries and were indistinguishable from control XX ovaries in that they expressed ovary-specific markers, and did not express Sertoli cell or Leydig cell-specific markers. These data indicate that the testis-determining pathway is interrupted at a very early stage in these XY gonads.

In addition to defects in testis differentiation, we found that double heterozygous *Wt1*+/−; *Sf1*+/− fetuses lacked adrenal glands and that neonates die shortly after birth, presumably from adrenal insufficiency. Because the gonads and adrenal cortex share a common origin [47], because the morphological defects we observed occur early in the development of these organs, and because the lethality and sex reversal phenotypes were sensitive to genetic background in a similar way, we hypothesize that the initial defect occurs during the adrenogonadal progenitor stage and that one or more *tda* gene(s) plays a role in the differentiation of this progenitor into the adrenal cortex and gonads. While it has been known that *Wt1*, *Sf1* and *Wnt4* are necessary for adrenal cortex differentiation [14]–[17], [34], [35], [48], our results show that *Wt1* and *Sf1* interact genetically during adrenal development. These results are consistent with results reported for fetuses that are double heterozygous for *Sf1* and *Cited2*, a transcription cofactor that interacts with *Wt1* [49]. Given that *Wt1* is thought to directly activate *Sf1* transcription in the gonadal progenitor, our results suggest that this genetic pathway is important for the differentiation of the adrenogonadal progenitor. It will be interesting to determine how *Wnt4* fits into this genetic pathway in the adrenogonadal progenitor.

### *Wt1* and *Sf1* regulate *Sry* expression, but reduced *Sry* transcript levels are not solely responsible for sex reversal in *Wt1+/−* or *Sf1+/−* B6 XY mice

Consistent with these indications of an early defect in testis differentiation, we found that *Sry* expression was markedly reduced in heterozygous mutant gonads. Our unpublished data indicate that reduced expression results from both a direct effect on *Sry* expression and an indirect effect on the differentiation of pre-Sertoli cells (manuscript in preparation). Interestingly, increasing *Sry* dosage by replacing the Y^AKR^ Chr in *Wt1*+/− or *Sf1*+/− B6 XY^AKR^ fetuses with the Y^AKR,*Sxr*^ Chr dramatically increased the amount of testis tissue that developed but cord differentiation remained somewhat abnormal. Our previous experiments showed that the *Sry^AKR^* and *Sry^Sxr^* alleles are expressed with similar profiles and at similar levels in gonads from B6 XY^AKR,*Sxr*^ fetuses [29], suggesting that *Sry* expression is essentially doubled in these gonads. Thus, testicular cord differentiation was compromised in *Wt1* and *Sf1* heterozygotes, regardless of *Sry* dosage. These results suggest that reduced *Sry* transcript levels are not the sole cause of sex reversal in *Wt1*+/− and *Sf1*+/− B6 XY^AKR^ mice and that the expression of critical genes other than *Sry* is likely to be affected. We consistently found that the *Sf1* heterozygous phenotype was more severe than the *Wt1* heterozygous phenotype. However, the reduction in *Sry* expression was not statistically different between the two heterozygotes, which supports our hypothesis that genes and cellular processes other than those controlled by SRY contribute to the phenotypic differences. Our result agree with previous reports suggesting that *Sry* expression levels alone do not predict whether a given Y Chr causes testis differentiation defects on the B6 genetic background [50] and that sex reversal is caused by both the presence of particular SRY protein isoforms found in *M. domesticus* mice and reduced *Sry* expression [29].

### Analysis of *Wt1* and *Sf1* heterozygous gonads identifies potential downstream targets

The analysis of marker gene expression using WISH provided insights into potential *Wt1* and *Sf1* target genes. For example, *Wnt4* expression was reduced in *Wt1*+/− and *Sf1*+/− B6 XX ovaries compared to +/+ B6 XX ovaries indicating that *Wt1* and *Sf1* might regulate *Wnt4* expression in ovaries (Figure 2). In contrast, *Wnt4* expression was elevated in *Wt1*+/− and *Sf1*+/− B6 XY^AKR^ gonads compared to +/+ gonads, but we suspect that this increase is due to the presence of more ovarian tissue in these gonads. Expression of *Wnt4* also was reduced in *Wt1*+/− XX and XY mesonephroi. Because *Sf1* is not expressed in mesonephroi, reduced *Wnt4* expression indicates in this tissue WT1 regulates *Wnt4* expression independently of SF1. *Ptch1* expression also was reduced in *Wt1*+/− XX and XY mesonephroi. Interestingly, *Ptch1* is strongly expressed in the region where the mesonephric tubules form and the number of tubules is reduced in *Wt1*−/− mesonephroi [14]. This result suggests that *Wt1* regulates *Ptch1* expression and that *Ptch1* could have a role in the formation of mesonephric tubules. However, these WISH results, though quite intriguing, require confirmation with a more quantitative method such as qRT-PCR.

### Co-expression of ovarian and testicular markers suggests reduced antagonism of the ovary and testis differentiation pathways in B6 XY gonads

Analysis of E13.5 B6 XY^AKR^ gonads showed that the anterior and posterior regions that lacked testicular cords expressed multiple ovarian somatic cell markers and that the germ cells within these domains had initiated meiosis. Therefore, these regions were not simply undifferentiated, but rather had initiated the ovary differentiation program in both somatic and germ cells. By E15.5, testicular cords are present throughout B6 XY^AKR^ gonads and it is unclear what happens to the ovarian regions between these two stages. Two possibilities are that these ovarian cells might transdifferentiate into the analogous testicular cell types, or regress via apoptosis. A recent analysis showed that the ovarian regions in *Sox9* transgenic XX ovotestes regress via apoptosis [51] and our preliminary analyses suggest that there is an increased number of apoptotic cells within the ovarian regions in B6 XY^AKR^ ovotestes (data not shown). In these regions it was clear that most cells expressed either ovarian (FOXL2) or testicular (AMH) markers and that these cells could be interspersed. We propose that co-expression of ovarian and testicular markers in E13.5 B6 XY ovotestes suggests that antagonism between these opposing differentiation pathways is reduced in these XY gonads. Contrary to results reported by Bradford and colleagues [52], we did observe a few cells at the boundaries between testicular and ovarian domains that appeared to express both granulosa and Sertoli cell markers. We could not unequivocally demonstrate co-expression using the markers and techniques presented here, however, the possibility that cells expressing both female and male supporting cell progenitor markers might be present during fetal gonad differentiation warrants further investigation.

### Why are the *Wt1* and *Sf1* B6 alleles haploinsufficient?

To determine why the *Wt1^B6^* and *Sf1^B6^* alleles are haploinsufficient and function as hypomorphs, we compared the coding sequences and expression levels of the B6 and D2 alleles. The results indicated that the *Wt1^B6^* and *Wt1^D2^* allele encode the same protein and that the ±KTS *Wt1* isoform expression ratio was the same in B6 and D2 gonad/mesonephros complexes. However, we found that *Wt1* expression throughout the initial stage of testis differentiation was lower in complexes from B6 fetuses, suggesting that in the B6 genetic background the *Wt1^B6^* allele is an expression hypomorph. In contrast, we were unable to detect significantly lower *Sf1* expression in complexes from B6 fetuses but we did identify one coding polymorphism in *Sf1*, suggesting that the *Sf1^B6^* allele is haploinsufficient not because its expression is reduced but possibly because the SF1^B6^ protein variant has reduced function. It appears that two SF1 variants are present among the common inbred mouse strains: SF1^A172^ (present in B6) and SF1^S172^ (present in D2). However, we found that homozygosity for the *Sf1* ^A172^ allele in F1 hybrids was not sufficient to cause sex reversal in the presence of the Y^POS^ Chr [as in (NZB/BlNJ×B6 XY^POS^) F1s] and that trans-heterozygosity for the *Sf1* ^A172^ and *Sf1* ^S172^ alleles was not sufficient to prevent sex reversal in the presence of the Y^POS^ Chr [as in (BALB/cBy×B6 XY^POS^) F1s]. These data show that the SF1^A172^ polymorphism alone does not account for sex reversal in the presence of the Y^POS^ Chr. Therefore, if the SF1^A172^ protein variant does indeed have reduced function during testis determination, then homozygosity for B6 alleles at other loci likely is necessary to cause *Sf1^B6^* to function as a hypomorph. Frigeri and colleagues [41] demonstrated that the SF1^A172^ and SF1^S172^ protein variants did not consistently differ in their ability to transactivate reporter genes containing tandem SF1 binding sites, *in vitro*. However, further experiments are needed to determine if the SF1^A172^ protein variant has reduced function during gonad differentiation *in vivo*.

### The *Wnt4* B6 allele is dosage-sensitive in the B6 genetic background and may contribute to hyperactivation of the ovary differentiation pathway

Our genetic results suggest that the *Wnt4^B6^* allele functions as a hypermorph in the ovary determination pathway. To ascertain if the ovary determination pathway was hyperactive in B6 vs. D2 gonads we quantified the expression of eight ovary-biased or ovary-specific genes in E12.5 B6 vs. D2 XX ovaries. We chose this developmental stage because existing data suggested that the ovary pathway would be active and that the chosen markers would be expressed at detectable levels. We chose to analyze XX and not XY gonads because an increase in ovary gene expression in XY gonads could be caused by failure to activate the testis pathway and subsequent failure to suppress the ovary pathway. We found that the expression of *Wnt4*, and four other genes was higher in B6 ovaries, indicating that the ovary determination pathway is activated earlier and/or to a greater degree in B6 vs. D2 ovaries. These results are consistent with those reported in an eQTL study of B6 vs. 129 gonads [53]. The B6 vs. D2 expression differences do not appear to be caused by gross differences in development between these two strains because we did not detect differences in limb morphology or in the size of the gonads or mesonephroi at the 27–28 ts stage used for these analyses (data not shown). However, not all eight genes were up-regulated in B6 ovaries: two were not significantly different, and one was down-regulated. We find it intriguing that *Rspo1*, which maps close to *Wnt4* within the *tda1* region, had reduced expression in B6 ovaries. However, because the *Rspo1* and *Wnt4* knockout phenotypes are very similar in that ovary and not testis differentiation is primarily affected, and because *Wnt4* expression is greatly reduced in *Rspo1*−/− XX gonads [54], [55], we think it is unlikely that *Rspo1* is *tda1* because we would expect that a strain with reduced *Rspo1* expression like B6 would be less sensitive to XY sex reversal, whereas B6 is, in fact, more sensitive. We note that for *Rspo1* no SNPs that differ between the B6 and D2 strains are present in dbSNP (Build 128), indicating that the RSPO1 protein does not differ between these strains. Furthermore, we suggest that the interaction between *Rspo1* and *Wnt4* may be indirect because *Rspo1* expression in decreased in B6 mice while *Wnt4* expression is increased. Nevertheless, these data do not formally exclude *Rspo1* as a *tda1* candidate.

### Are *Wt1*, *Sf1*, and *Wnt4 tda* genes?

More than twenty-five years ago, two of the authors hypothesized that the B6-derived *tda* genes could function later than normal in the testis determination pathway and/or, because the testis and ovary determination pathways antagonize each other, could function earlier than normal in the ovary determination pathway [25], [56]. Thus, if a *tda* gene functions in the testis-determining pathway, then reducing its dosage in B6 XY mice should cause sex reversal, whereas reducing its dosage in D2 XY mice should not. Similarly, if a *tda* gene functions in the ovary-determining pathway and antagonizes testis differentiation, then reducing its dosage should ameliorate B6 XY sex reversal. The *Wt1* and *Sf1* genes fulfill the criteria as late functioning testis-determining genes and *Wnt4* fulfills the criteria as an early function ovary-determining gene that antagonizes testis differentiation. Furthermore, our results show that B6 XY^POS^, *Wt1*+/− B6 XY^AKR^, and *Sf1*+/− B6 XY^AKR^ sex reversal share similar gonadal phenotypes and that these three sex reversals are sensitive to genetic background in a similar way. Additionally, we have recently determined that a major modifier of *Sf1*+/− B6 XY^AKR^ sex reversal that differs between the B6 and D2 genomes, maps to the distal region of Chr 4, which suggests that the *tda1* modifier gene plays a prominent role in both *Sf1*+/− B6 XY^AKR^ and B6 XY^POS^ sex reversal (manuscript in preparation). However, while our data are consistent with the hypothesis that *tda2* is comprised of *Wt1* and *Sf1* and that *tda1* is *Wnt4*, the genetic intervals defined by the linkage analyses are fairly broad, and further experiments are needed to directly test this hypothesis and to exclude the possibility of other candidate genes in the *tda1* and *tda2* intervals.

Undoubtedly, *tda* genes in addition to *tda1* and *tda2* contribute to B6 XY^POS^ sex reversal because, as shown in the original linkage analysis, some XY^POS^ fetuses that were homozygous for B6 alleles at the *tda1* and *tda2* loci contained normal appearing testes [24]. Nevertheless, it appears that *tda1* and *tda2* play an important role in many cases of B6 XY sex reversal because we have mapped a major modifier of *Dax1* −/Y sex reversal that differs between the B6 and D2 genomes to the same chromosome region as *tda1* [22], and have recently determined that major modifiers of *Gata4* +/− B6 XY^AKR^ sex reversal [23] map to the same chromosome regions as *tda1* and *tda2* (manuscript in preparation).

### An additive model to explain B6 XY sex reversal

Overall, our data suggest that the B6 inbred strain is sensitive to XY sex reversal, at least in part, due to hypomorphic alleles of *Wt1* and/or *Sf1* that compromise the testis determination pathway, as well as a hypermorphic allele of *Wnt4* that antagonizes testis development. We suggest that in XY gonads of most inbred strains, such as DBA/2J, the testis determination pathway reaches a critical threshold that is sufficient to inactivate the ovary determination pathway prior to the developmental stage when the ovary determination pathway reaches a threshold that is sufficient to inactivate the testis determination pathway. In contrast, in a few inbred strains such as B6, the testis determination pathway is compromised and the ovary determination pathway is hyperactivated, thus the interval between the developmental stages at which these two thresholds are reached is greatly reduced. We propose that this interval is even further reduced or eliminated in B6 mice carrying certain *M. domesticus*-derived *Sry* alleles that do not efficiently activate the testis determination pathway, thus allowing ovotestes to develop transiently in B6 XY^AKR^ mice and allowing stable ovotestes and ovaries to develop in B6 XY^POS^ mice, for example. In summary, we propose that sex reversal in B6 XY^POS^ gonads is the result of the additive effects of 1) hypomorphic *Wt1^B6^* and/or *Sf1^B6^* alleles, 2) a hypermorphic *Wnt4^B6^* allele and hyperactivation of the ovary pathway, 3) reduced function of *M. domesticus*-derived SRY proteins, 4) reduced expression of the *Sry^POS^* allele, and 5) the presence of other unidentified *tda* genes.

### Mouse models of human dosage-sensitive sex reversal

These studies demonstrate that *Wt1*, *Sf1*, and *Wnt4* are dosage-sensitive in mice, as they are in humans, and show that this effect is modulated by genetic background, as we suspect it is in humans. They also demonstrate that the *Wt1+/−* and *Sf1+/−* B6-Y^AKR^ mice are good models for the corresponding human XY sex reversals involving heterozygous mutations in these genes. We suggest that these heterozygous mice will be very useful for the elucidation of *Wt1* and *Sf1* functions during gonad differentiation because the heterozygous phenotype is far less severe than the homozygous phenotype, which is a complete abrogation of gonad differentiation and subsequent apoptosis at the genital ridge stage. Thus, we think it is likely that these strains will be particularly useful in providing further insights into the roles of *Wt1* and *Sf1* in organogenesis and human disease.

## Materials and Methods

### Animals and genotyping

Null (knockout) alleles of *Wt1* (*Wt1^tm1Jae^*) [14], *Sf1* (*Nr5a1^tm1Klp^*) [16], and *Wnt4* (*Wnt4^tm1Amc^*) [34] were transferred to the B6 and D2 inbred strain backgrounds using standard backcrossing. No sex ratio disturbances were noted in heterozygous progeny during construction of these congenic strains. Experimental results reported here were conducted after backcross generation N10 or greater was achieved. The B6-Y^POS^, B6-Y^AKR^, B6-Y^AKR,*Sxr*^ and D2-Y^AKR^ consomic strains were previously described [19], [20], [23], [29], [57]. This study was carried out in strict accordance with the recommendations in the Guide for the Care and Use of Laboratory Animals of the National Institutes of Health. The Jackson Laboratory and Boston University Laboratory Animal Science Center are AALAS accredited and all animal procedures were approved by The Jackson Laboratory Animal Care and Use Committee (protocols LAH:93-26, LAH:95-01, LAH:95-02) or the Boston University Animal Care and Use Committee (protocol AN-13935).

The presence of a *Wt1*, *Sf1*, or *Wnt4* knockout allele was determined by multiplex PCR using primers to detect the neomycin cassette and the myogenin gene as a control [23]. In crosses involving multiple knockout alleles, gene-specific PCR assays were used to amplify both the normal and the targeted alleles [46], [58]. The genetic sex of fetuses was determined by PCR using Y Chr specific primers and myogenin control primers [59]. The B6-Y^AKR,*Sxr*^ strain carries a rearranged Y Chr containing two *Sry* alleles: the *M. domesticus*-derived *Sry* ^AKR^ allele and the *M. musculus*-derived *Sry^Sxr^* allele. The presence of the *M. domesticus* and/or *M. musculus Sry* alleles was determined by PCR followed by digestion with *Nla*IV restriction enzyme using primers that flank a polymorphism within the *Sry* gene [29].

### Embryo isolation and staging, and determination of sexual phenotype

Timed matings were used for all embryonic experiments. Noon on the day of vaginal plug detection was designated as E0.5. Embryos between E10.5 and 12.5 were precisely staged by counting the number of tail somites (E10.5∼8 ts, E11.5∼18 ts, E12.5∼30 ts) [60] and those between E12.5 and E16 were staged by limb morphology and the extent of cranial vessel formation [61].

To classify the gonads of individual E14.5–16 fetuses, the gonads were removed from the body cavity and immediately examined with the aid of an inverted microscope using transmitted light, and then photographed [62]. Testes were identified as having distinctive cords and vasculature, and an incipient tunica albuginea. Ovaries were identified as having a reticular appearance and lacking the structures noted above in the testes. Ovotestes contained a region with a reticular appearance at one or both ends of the gonad with the remainder containing cords. The sexual phenotype of weaning age (∼3 weeks postpartum) mice was determined by examining the external genitalia, noting the anal-genital distance, and observing whether mammary gland-associated yellow-pigmented hairs were present [63]. Gonads from E13.5 fetuses were classified at the time of dissection, when the observer was blind to the fetal genotype. Gonads without any testicular cords were classified as ovaries, those with testicular cords only in the central region of the gonad were classified as ovotestes, and those with testicular cords throughout were classified as testes. Those with disorganized testicular cords throughout the gonad were classified as abnormal testes.

### Whole-mount immunohistochemistry and RNA *in situ* hybridization

Gonad/mesonephros complexes were fixed overnight in 4% paraformaldehyde in PBS at 4°C. Whole-mount indirect fluorescent immunohistochemistry (WIHC) was performed essentially as previously described [64], except that a blocking buffer containing 3% BSA, 10% donkey serum, 0.1% Triton-X, 0.02% sodium azide in PBS was used when the anti-PDGFRa or anti-MIS (AMH) antibodies were employed. Primary antibodies and dilutions are listed in Table S1. Secondary antibodies were donkey anti-IgG conjugated with Cy3 or Cy5 (Jackson ImmunoResearch, 1∶500) or AF488 (Molecular Probes, 1∶750). Samples were imaged using a Zeiss LSM510 confocal microscope.

Standard protocols for whole-mount *in situ* hybridization (WISH) were used with minor changes [46], [65]. Gene-specific probes are listed in Table S1. The *Rec8* probe was synthesized from a full-length cDNA clone (IMAGE ID:6335959, GenBank: BC052155.1). Antisense and sense probes were tested in parallel, and only signal specific to the antisense probes was reported as gene-specific expression. Each result reported was replicated in a minimum of four samples.

### RNA extraction, reverse transcription, and quantitative RT–PCR

Total RNA was isolated using the RNeasy mini kit (Qiagen). Samples were treated with Turbo DNase (Ambion) and subsequently treated with DNase inactivation reagent using the DNA-free protocol (Ambion). RNA Samples were determined to be free of DNA by PCR using primers specific for the *Myog* or *Lhx1* genes [29]. cDNA was synthesized using oligo-dT primers and reverse transcriptase (RT). The RT reaction was tested for the presence of intact cDNA by PCR amplification with primers specific for the *Hprt* transcript [46].

Relative *Sry* expression in mutant vs. normal gonad/mesonephros complexes was determined using TaqMan Universal PCR Master Mix, and *Sry* (Mm00441712_s1) and *Gapdh* (Mm99999915_g1) Assays-on-Demand following the manufacturer's standard protocol (Applied Biosystems). Relative *Wt1* and *Sf1* expression in B6 vs. D2 gonad/mesonephros complexes was determined using *Wt1* (Mm00460570_m1), *Sf1*(Mm00446826_m1) and *Gapdh* TaqMan Assays-on-Demand. Relative expression of ovary pathway genes in B6 vs. D2 ovaries was determined using Power SYBR Green PCR Master Mix (Applied Biosystems) and the following primers: *Adamts19* (5′-CCAGATGCCTCCTGCTTTTA and 5′-GGTGCGGGTGACCTATGAT: 165 bp product), *Wnt4* (5′-AGGATGCTCGGACAACATCG and 5′-CGCATGTGTGTCAAGATGGC: 149 bp product), *Igfbp2* and *1700106J16Rik* as in [46], *Fst*, *Bmp2*, and *Hprt* as in [44], and *Irx3* as in [38].

Quantitative RT-PCR (qRT-PCR) assays were performed on an ABI PRISM 7900HT Sequence Detection System using SDS 2.3 software to determine the average cycle threshold (Ct) value from three technical replicates for each sample. Average Ct values for *Hprt* or *Gapdh* were subtracted from the average Ct values for each gene for each sample, to determine the normalized average Ct value (ΔCt). When expression was profiled across developmental stages, the results are represented as expression relative to the endogenous control gene (2^−ΔCt^), when expression was compared within a single developmental stage, the results were determined by subtracting the average ΔCt for D2 ovaries according to the 2^−ΔΔCt^ method [66]. In pairwise comparisons of gene expression levels with *a priori* predictions about the direction of the effect, such as detecting lower expression of *Wt1* and *Sf1* or higher expression of *Wnt4* and other ovarian genes in B6 samples, statistically significant differences were identified by one-tailed unpaired Student's t-tests (α = 0.05) using Microsoft Excel. For statistical analysis of *Sry* expression, one-way ANOVA was used to test for a significant effect of genotype in multiple comparisons and two-tailed unpaired Student's t-tests (α = 0.05) in pairwise comparisons within a developmental stage using JMP version 8 software (SAS Institute Inc.).

Multi-gene quantitative real time RT-PCR was conducted using RNA extracted from E12 gonad-mesonephros complexes and E14 gonads as described in [44]. Only E14 XY^AKR^ mutant gonads lacking any testicular cords (classified as ovaries) were used for this analysis.

### Genotyping assay for the presence of *Sf1^A172^* versus *Sf1^S172^* alleles

The presence of the *Sf1^A172^* and *Sf1^S172^* alleles was determined using a previously published genotyping assay [41]. Briefly, a region of *Sf1* exon 3 including codon 172 was amplified from genomic DNA using 5′-TGCGTGCTGATCGAATGC and 5′-CCAGTCGACAATGGAGATAAAGG as primers and the PCR product digested using *Nco*I. Genomic DNA containing the *Sf1^A172^* allele contains a *Nco*I recognition site resulting in two fragments of 331 and 262 bp, while the *Sf1^S172^* allele lacks the *Nco*I recognition site resulting in an undigested 593 bp fragment. Genomic DNA was obtained from mice in the Eicher Laboratory mouse colony or purchased from the JAX Mice DNA Resource [C58/J (000669), NZB/BlNJ (000684), C3H/HeSnJ (000661), BALB/cBY (000650), SM/J (000687)].

## Supporting Information

Figure S1 Transmitted light micrographs illustrating the phenotypic range of *Wt1*+/− B6 XY^AKR^ E15.5 gonads. Only *Wt1*+/− gonad/mesonephros complexes are shown because *Wt1*+/− and *Sf1*+/− B6 XY^AKR^ gonads had similar morphology. All images at 10× magnification, cranial is to the left, and the gonad is above the dotted black line with the mesonephros below it. Heterozygous XY^AKR^ ovaries (C) did not have testicular cords, lacked obvious structure including the coelomic vessel, and were more similar in size to age-matched +/+ XX ovaries (A) than +/+ XY^AKR^ testes (B). Heterozygous XY^AKR^ ovotestes (D) usually had central regions with testicular cords (outlined with dotted white lines) and regions at the cranial (anterior) and caudal (posterior) poles that lacked obvious structure and appeared to be ovarian. The regions with cords were associated with a coelomic vessel (arrowhead) whereas this vasculature was not present in the flanking regions. The ovary in (C) and the ovotestis in (D) were present in the same fetus. Some heterozygous XY^AKR^ testes were slightly abnormal (E) in that they were smaller than +/+ B6 XY^AKR^ testes and appeared to have delayed testis cord formation. However, these did not contain obvious ovarian tissue. In contrast, some +/− B6 XY^AKR^ testes had well-developed cords throughout (F) and were very similar to +/+ B6 XY^AKR^ testes. The gonads in (A, B, E, and F) were dissected from fetuses from the same litter.
(TIF)

Figure S2 Expression of ovary-specific markers in B6 XY^AKR^ E13.5 gonads analyzed by WIHC and WISH. A) WIHC analysis. FOXL2, which is normally expressed in ovarian somatic cells, often was expressed in the poles of B6 XY^AKR^ gonads (left panel, 20× magnification). In some cases, FOXL2-expressing cells were found in regions containing AMH-expressing Sertoli cells and incipient testicular cords (right panel, 40× magnification). B) WISH analysis. The ovary-specific somatic cell marker *Irx3*, and meiotic germ cell markers *Stra8* and *Rec8* were expressed in the cranial (anterior) and/or caudal (posterior) poles of B6 XY^AKR^ gonads. When *Stra8* and *Rec8* were expressed at both poles in B6 XY^AKR^ gonads at this developmental stage, expression was higher at the cranial (left) vs. the caudal pole (right). The gonad/mesonephros complex in each panel is oriented with the gonad above the mesonephros and the anterior pole to the left.
(TIF)

Table S1 WIHC and WISH markers. Pertinent information on the antibodies and riboprobes used to analyze morphology and marker gene expression during fetal gonad differentiation.
(DOCX)
