# Supplementary figures and images for: Sex Reversal in C57BL/6J XY Mice Caused by Increased Expression of Ovarian Genes and Insufficient Activation of the Testis Determining Pathway
Source: PLoS Genet. 2012 Apr 5;8(4):e1002569. doi: 10.1371/journal.pgen.1002569 (PMC3320579; doi:10.1371/journal.pgen.1002569)

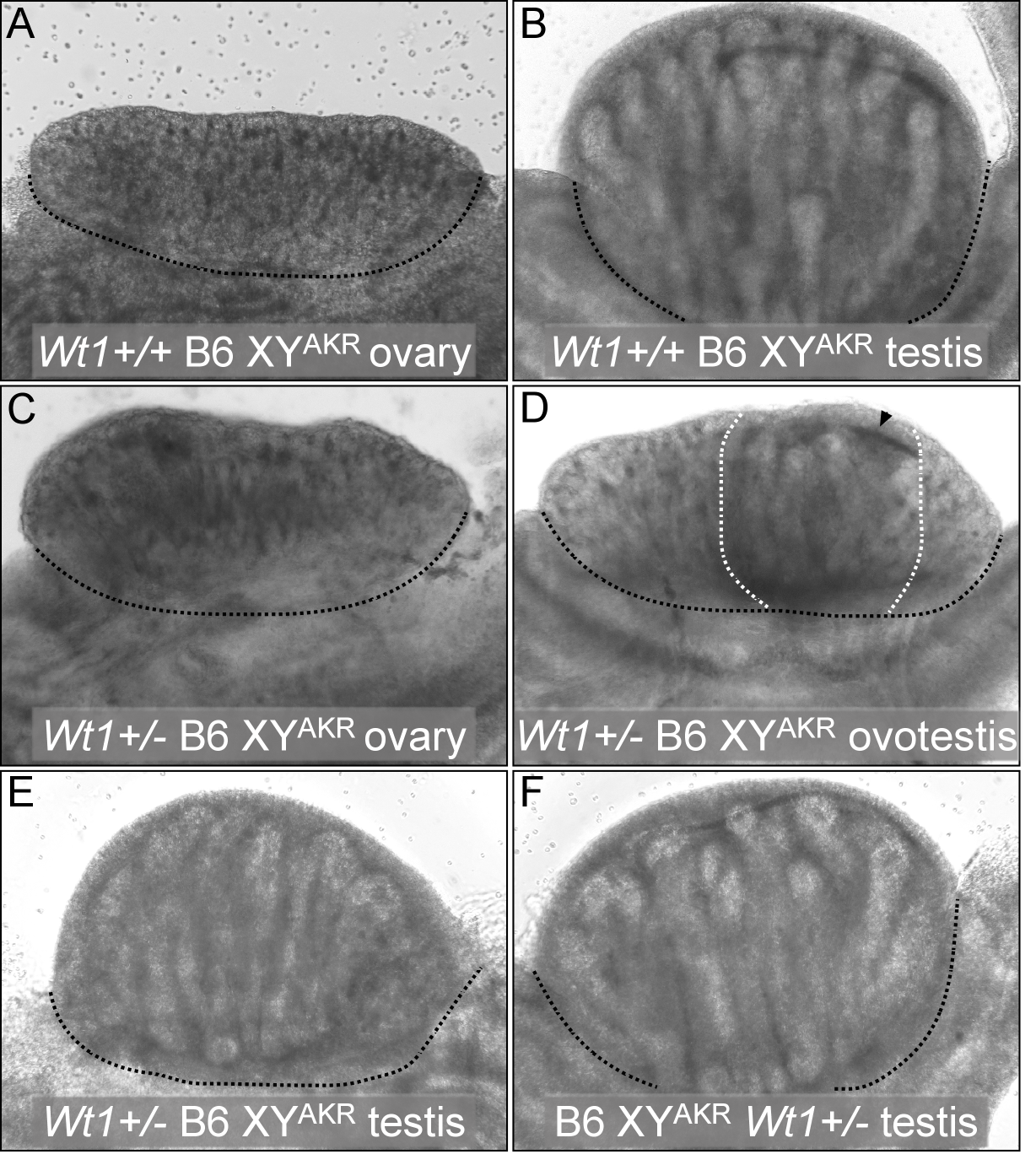

Supplement: Figure S1 — Transmitted light micrographs illustrating the phenotypic range of Wt1+/− B6 XYAKR E15.5 gonads. Only Wt1+/− gonad/mesonephros complexes are shown because Wt1+/− and Sf1+/− B6 XYAKR gonads had similar morphology. All images at 10× magnification, cranial is to the left, and the gonad is above the dotted black line with the mesonephros below it. Heterozygous XYAKR ovaries (C) did not have testicular cords, lacked obvious structure including the coelomic vessel, and were more similar in size to age-matched +/+ XX ovaries (A) than +/+ XYAKR testes (B). Heterozygous XYAKR ovotestes (D) usually had central regions with testicular cords (outlined with dotted white lines) and regions at the cranial (anterior) and caudal (posterior) poles that lacked obvious structure and appeared to be ovarian. The regions with cords were associated with a coelomic vessel (arrowhead) whereas this vasculature was not present in the flanking regions. The ovary in (C) and the ovotestis in (D) were present in the same fetus. Some heterozygous XYAKR testes were slightly abnormal (E) in that they were smaller than +/+ B6 XYAKR testes and appeared to have delayed testis cord formation. However, these did not contain obvious ovarian tissue. In contrast, some +/− B6 XYAKR testes had well-developed cords throughout (F) and were very similar to +/+ B6 XYAKR testes. The gonads in (A, B, E, and F) were dissected from fetuses from the same litter. (TIF) [file pgen.1002569.s001.tif]

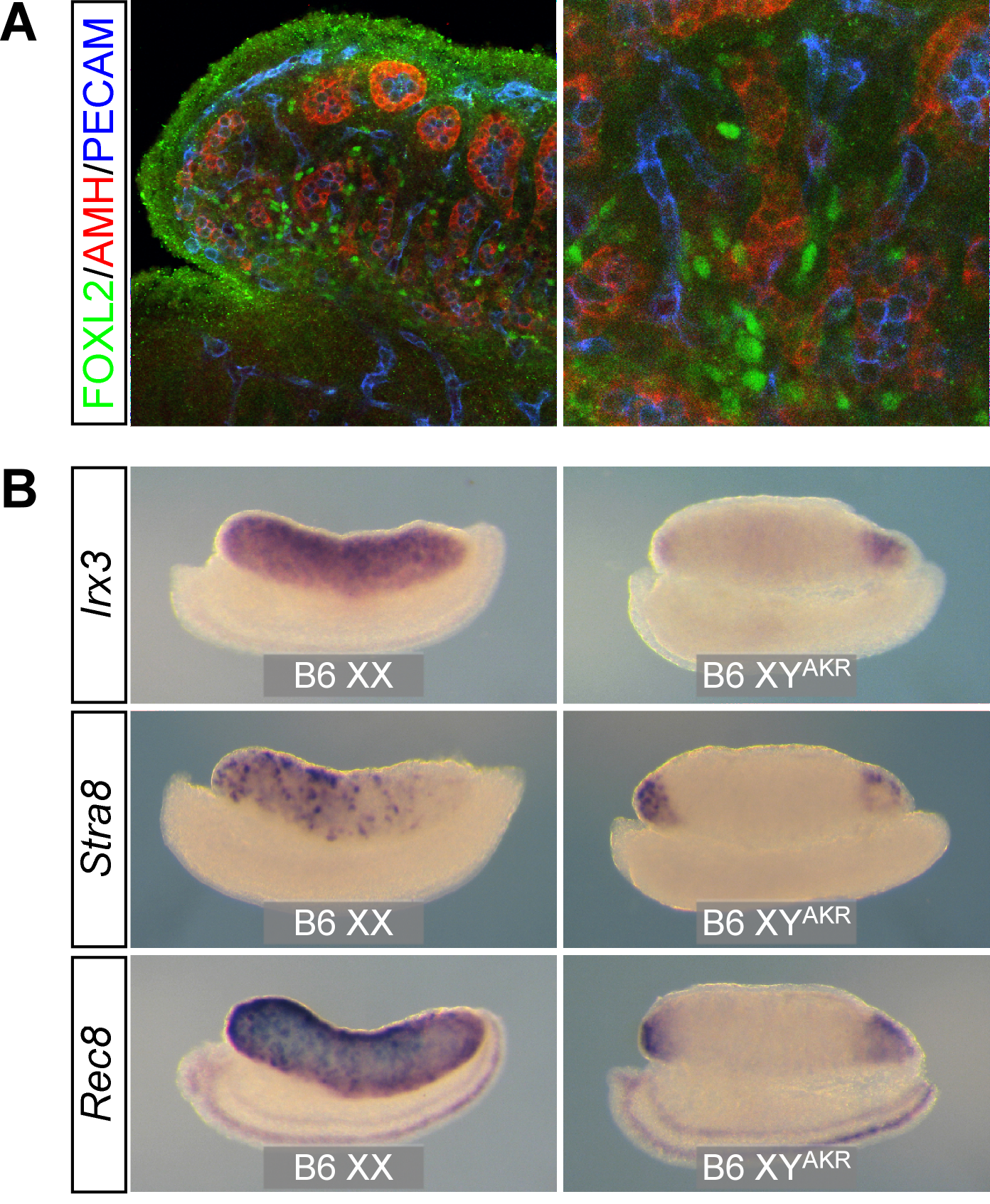

Supplement: Figure S2 — Expression of ovary-specific markers in B6 XYAKR E13.5 gonads analyzed by WIHC and WISH. A) WIHC analysis. FOXL2, which is normally expressed in ovarian somatic cells, often was expressed in the poles of B6 XYAKR gonads (left panel, 20× magnification). In some cases, FOXL2-expressing cells were found in regions containing AMH-expressing Sertoli cells and incipient testicular cords (right panel, 40× magnification). B) WISH analysis. The ovary-specific somatic cell marker Irx3, and meiotic germ cell markers Stra8 and Rec8 were expressed in the cranial (anterior) and/or caudal (posterior) poles of B6 XYAKR gonads. When Stra8 and Rec8 were expressed at both poles in B6 XYAKR gonads at this developmental stage, expression was higher at the cranial (left) vs. the caudal pole (right). The gonad/mesonephros complex in each panel is oriented with the gonad above the mesonephros and the anterior pole to the left. (TIF) [file pgen.1002569.s002.tif]
