# Supplementary material for: Sex Reversal in C57BL/6J XY Mice Caused by Increased Expression of Ovarian Genes and Insufficient Activation of the Testis Determining Pathway
Source: PLoS Genet. 2012 Apr 5;8(4):e1002569. doi: 10.1371/journal.pgen.1002569 (PMC3320579; doi:10.1371/journal.pgen.1002569)
Supplement: Table S1 — WIHC and WISH markers. Pertinent information on the antibodies and riboprobes used to analyze morphology and marker gene expression during fetal gonad differentiation. (DOCX) [file pgen.1002569.s003.docx]

**Table S1. WIHC and WISH markers.**

| Marker | Type | Dilution | Gonadal cell type identified at E13.5 | Source/Reference |
| --- | --- | --- | --- | --- |
| WIHC |  |  |  |  |
| SOX9 | rabbit polyclonal | 1:1000 | Sertoli cells | [1] |
| AMH/MIS | goat polyclonal | 1:200 | Sertoli cells | Santa Cruz Biotechnology |
| FGFR2 | rabbit polyclonal | 1:500 | ovary: surface of somatic cells  testis: Sertoli cell nucleus | Santa Cruz Biotechnology |
| PDGFRa | goat polyclonal | 1:500 | interstitial cells of ovary and testis, coelomic epithelium of testis | R&D Systems |
| SF1 | rabbit polyclonal | 1:1000 | Sertoli and Leydig cells | [2] |
| FOXL2 | rabbit polyclonal | 1:400 | granulosa cells | [3] |
| GATA4 | goat polyclonal | 1:400 | somatic cells except vascular endothelium | Santa Cruz Biotechnology |
| PECAM/  CD31 | rat monoclonal | 1:300 | germ and vascular endothelial cells | BD Pharmingen |
|  |  |  |  |  |
| WISH |  |  |  |  |
| *Wnt4* |  |  | ovary somatic cells | [4] |
| *Fst* |  |  | ovary somatic cells | [5] |
| *Irx3* |  |  | ovary somatic cells | [6] |
| *Stra8* |  |  | ovary pre-meiotic germ cells | [5] |
| *Rec8* |  |  | ovary pre-meiotic germ cells | Open Biosystems |
| *Ptch1* |  |  | Leydig cells | [7] |

1. Gasca S, Canizares J, De Santa Barbara P, Mejean C, Poulat F, et al. (2002) A nuclear export signal within the high mobility group domain regulates the nucleocytoplasmic translocation of SOX9 during sexual determination. Proc Natl Acad Sci U S A 99: 11199-11204.

2. Morohashi K, Hatano O, Nomura M, Takayama K, Hara M, et al. (1995) Function and distribution of a steroidogenic cell-specific transcription factor, Ad4BP. J Steroid Biochem Mol Biol 53: 81-88.

3. Cocquet J, Pailhoux E, Jaubert F, Servel N, Xia X, et al. (2002) Evolution and expression of FOXL2. J Med Genet 39: 916-921.

4. Parr BA, Shea MJ, Vassileva G, McMahon AP (1993) Mouse Wnt genes exhibit discrete domains of expression in the early embryonic CNS and limb buds. Development 119: 247-261.

5. Menke DB, Koubova J, Page DC (2003) Sexual differentiation of germ cells in XX mouse gonads occurs in an anterior-to-posterior wave. Dev Biol 262: 303-312.

6. Jorgensen JS, Gao L (2005) Irx3 is differentially up-regulated in female gonads during sex determination. Gene Expr Patterns 5: 756-762.

7. Goodrich LV, Johnson RL, Milenkovic L, McMahon JA, Scott MP (1996) Conservation of the hedgehog/patched signaling pathway from flies to mice: induction of a mouse patched gene by Hedgehog. Genes Dev 10: 301-312.
